# The type IV pilus protein PilU functions as a PilT-dependent retraction ATPase

**DOI:** 10.1371/journal.pgen.1008393

**Published:** 2019-09-16

**Authors:** David W. Adams, Jorge M. Pereira, Candice Stoudmann, Sandrine Stutzmann, Melanie Blokesch

**Affiliations:** Laboratory of Molecular Microbiology, Global Health Institute, School of Life Sciences, EPFL-SV-UPBLO, Ecole Polytechnique Fédérale de Lausanne (EPFL), CH, Lausanne, Switzerland; Newcastle University, UNITED KINGDOM

## Abstract

Type IV pili are dynamic cell surface appendages found throughout the bacteria. The ability of these structures to undergo repetitive cycles of extension and retraction underpins their crucial roles in adhesion, motility and natural competence for transformation. In the best-studied systems a dedicated retraction ATPase PilT powers pilus retraction. Curiously, a second presumed retraction ATPase PilU is often encoded immediately downstream of *pilT*. However, despite the presence of two potential retraction ATPases, *pilT* deletions lead to a total loss of pilus function, raising the question of why PilU fails to take over. Here, using the DNA-uptake pilus and mannose-sensitive haemagglutinin (MSHA) pilus of *Vibrio cholerae* as model systems, we show that inactivated PilT variants, defective for either ATP-binding or hydrolysis, have unexpected intermediate phenotypes that are PilU-dependent. In addition to demonstrating that PilU can function as a *bona fide* retraction ATPase, we go on to make the surprising discovery that PilU functions exclusively in a PilT-dependent manner and identify a naturally occurring pandemic *V*. *cholerae* PilT variant that renders PilU essential for pilus function. Finally, we show that *Pseudomonas aeruginosa* PilU also functions as a PilT-dependent retraction ATPase, providing evidence that the functional coupling between PilT and PilU could be a widespread mechanism for optimal pilus retraction.

## Author summary

Bacteria interact with their surroundings using micrometre scale polymers called type IV pili. They allow bacteria to physically sense, attach and move on surfaces, and even to take up DNA. Consequently they represent important mechanisms of environmental survival and pathogenesis. The versatility of type IV pili is made possible by dedicated motors that power repeated cycles of extension and retraction. Curiously, although the ATPase PilT is well established as the retraction motor, many species have an additional PilT-like protein called PilU. However, how PilU functions has remained unclear, especially since when PilT is absent it is unable to take over its function. In this work we took a different approach. Instead of deleting *pilT*, we made inactivated variants and studied the functionality of two distinct types of pili used by the human pathogen *Vibrio cholerae* to survive in its natural aquatic environment. This allowed us to make the unexpected discovery that PilU is capable of acting as a retraction ATPase, but that it is not an independent motor and instead exerts its function via PilT. Our results suggest this functional coupling between PilT and PilU may be common in other bacteria.

## Introduction

Type IV pili (T4P) are a widespread class of cell surface polymers found throughout the bacteria and archaea [1–3]. In bacteria, they allow cells to physically sense and interact with the environment around them [4]. Consequently they play critical roles in environmental survival and pathogenesis. The type IVa pilus (T4aP) machinery responsible for pilus biogenesis is conserved and widely distributed [2, 5]. Briefly, individual pilin subunits are extracted from the membrane, polymerised into a filament composed primarily of a single major pilin, and guided across the cell envelope layers, before in Gram-negative bacteria exiting the cell surface via an outer membrane pore [6–9]. A unique feature of T4P is their ability to undergo repeated cycles of extension and retraction [10]. This affords considerable functional versatility. For example, cycles of extension, transient attachment, and retraction, powers a form of flagellum-independent motility known as twitching motility [11, 12]. Retraction also allows cells to sense and adhere to surfaces, to take up DNA during natural competence for transformation and is also exploited as an entry mechanism by some bacteriophages [2].

T4aP dynamics are orchestrated by dedicated extension (*e*.*g*. PilB) and retraction (*e*.*g*. PilT) ATPases [13, 14]. These proteins belong to the Additional Strand Catalytic ‘E’ (ASCE) subfamily of AAA+ ATPases [15, 16]. Both are cytoplasmic proteins that *in vitro* form oblong hexamers around a central pore [17–22]. They interface with the T4aP machinery via the platform protein PilC, which protrudes from the membrane at the base of the machine and likely sits inside the central pore, and are further clamped in place by PilM [7, 23, 24]. The observation that ATP-binding and subsequent hydrolysis leads to a series of conformational changes, which propagate around the pores of PilB and PilT hexamers in opposite directions [20–22, 25, 26], has led to a model whereby the rotation of PilC transduces the action of the ATPase [7, 22, 25, 26]. Accordingly, when PilB is engaged PilC rotates in a clockwise direction, leading to pilus extension. Conversely, when PilT is engaged, its pore rotates in a counter-clockwise direction, leading to pilus retraction. However, the exact details remain unclear and an alternative PilC-gating model has also recently been proposed [10].

Dedicated retraction ATPases are a common feature of T4aP systems [2, 5] and are thought to be required for generating the force needed to achieve pilus function [10]. Indeed, PilT is the strongest molecular motor known and has been studied extensively [27, 28]. In a range of different species (*e*.*g*. *Acinetobacter baylyi*, *Dichelobacter nodosus*, *Pseudomonas aeruginosa*, *Pseudomonas stutzeri*, *Neisseria gonorrhoeae*, *Neisseria meningitidis* and *Synechocystis* sp. PCC6803) the deletion of *pilT* results in a total loss of pilus function [29–37]. Although cells lacking *pilT* remain piliated, they are unable to mediate twitching motility or DNA-uptake, often exhibit altered surface adherence and are typically hyper-piliated. Notably, a second putative retraction ATPase, PilU, is often encoded directly downstream of *pilT* within an operon, or elsewhere on the genome, and like PilT has ATPase activity *in vitro* [13]. Interestingly, *Neisseria* sp. possess an additional PilT paralogue (PilT-2), and in some species as many as four PilT paralogues can be present [35, 38, 39]. As noted previously by Brown *et al*., it can be difficult to unify results across different organisms, in particular due to differences in assay conditions [35]. Nevertheless, compared to PilT, PilU often appears to be dispensable for T4aP function as its deletion produces either subtle phenotypes or defects only in specific functions [31, 33, 35, 37, 40–42]. One notable exception is that both PilT and PilU are required for twitching underneath agar in several species [32, 33, 40]. Likewise, both PilT and PilU are required for pathogenesis in a number of species [33, 42, 43].

*Vibrio cholerae* is an aquatic Gram-negative bacterium responsible for the pandemic human disease cholera. Strains representative of the on-going 7^th^ cholera pandemic utilise two distinct T4aP systems. First, mannose-sensitive haemagglutinin (MSHA) pili, which are produced constitutively under laboratory conditions, are required for surface sensing and attachment, are important for the initiation of biofilm formation, and are also a receptor for a filamentous bacteriophage [44–48]. Second, DNA-uptake pili are produced during growth upon chitinous surfaces, which are abundant in the aquatic environment [46, 49, 50]. These pili are highly dynamic and retract to take up DNA during natural competence for transformation [50–52]. Moreover, DNA-uptake pili bind to chitinous surfaces and are required for chitin colonization under flow [52]. Notably, PilT function is shared between these two different T4aP systems, and, as in other species, deletion of *pilT* leads to a total loss of pilus function. This renders MSHA pili defective for surface sensing, attachment, and biofilm formation [47, 53]. Similarly, DNA-uptake pili loose their rapid dynamics, become hyper-piliated and are unable to mediate transformation [50–52]. Furthermore, DNA-uptake pili can also interact with one another in a sequence specific manner, which in liquid culture results in the auto-aggregation of retraction-deficient cells [52]. In contrast, PilU, which is encoded directly downstream of *pilT*, has no apparent effect on the function of either system [47, 50, 52]. The dispensability of PilU for T4aP function is in itself not surprising, since PilT remains available to mediate retraction. However, an enduring question has been why PilU is unable to take over in the absence of PilT, especially given the similarity between the two proteins.

Here, using the two distinct T4aP of *V*. *cholerae* as model systems we have used a genetic approach to investigate the relative contributions of PilT and PilU. Unexpectedly, PilT variants engineered to be non-functional had only intermediate phenotypes, revealing that when PilT is inactivated PilU is indeed capable of taking over and functioning as a *bona fide* retraction ATPase. However, we go to demonstrate that PilU itself is not a separate retraction motor but functions exclusively in a PilT-dependent manner. We provide evidence that this functional coupling is likely a conserved feature of PilU.

## Results and discussion

### Multiple phenotypes depend on the retraction ATPase PilT

**Fig 1:**
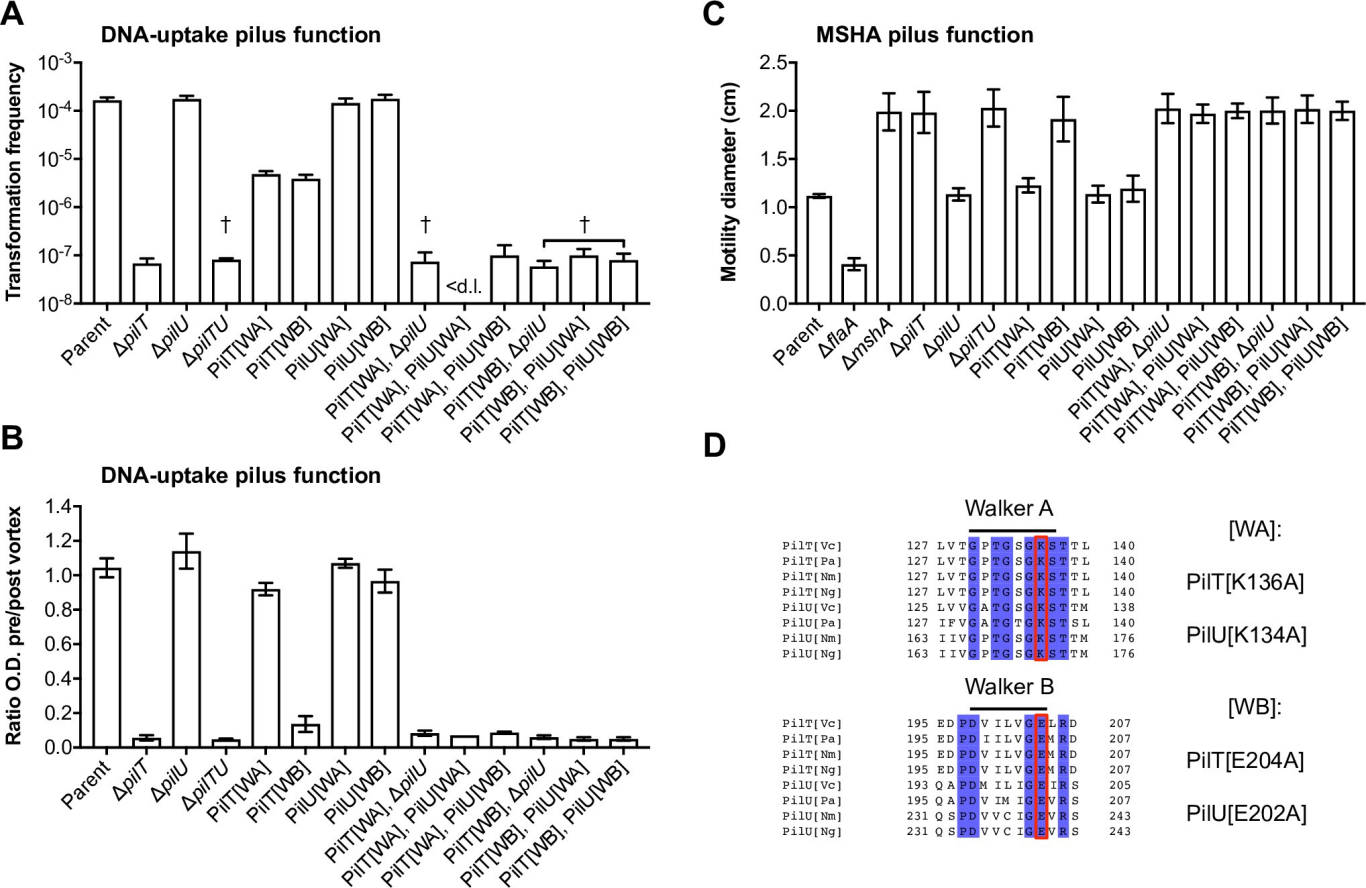
Inactivated PilT variants reveal a role for PilU in DNA-uptake pilus and MSHA pilus function. (**A**-**C**) The role of PilT and PilU, and their corresponding Walker A and Walker B variants, was assessed using (**A**) natural transformation and (**B**) auto-aggregation as readouts for DNA-uptake pilus function, and (**C**) by swarming motility as a readout for MSHA pilus function. All strains contain an arabinose-inducible copy of *tfoX* (*araC P*_BAD_-*tfoX*; Tn*tfoX*), within an ectopically integrated transposon, which was used for chitin-independent competence induction in A and B. (**A**) Chitin-independent transformation assay. Transformation frequencies are the mean of three repeats (+S.D.). < d.l., below detection limit. †, < d.l. in one repeat. (**B**) Aggregation is shown as the ratio of the culture optical density (O.D. _600 nm_) before and after vortexing in the presence of *tfoX* induction. Values are the mean of three repeats (±S.D.). Values close to 1 = No aggregation. Values close to 0 = Full aggregation. (**C**) Surface motility was determined on soft LB agar plates. The swarming diameter (cm) is the mean of three repeats (±S.D.). A flagellin-deficient (Δ*flaA*) non-motile strain was used as a negative control. (**D**) Alignments highlighting the conserved Walker A and Walker B motifs of PilT and PilU. Protein sequences were aligned using Clustal Omega and the figure prepared using Jalview. Identical residues are shaded in blue. Residues targeted for mutagenesis in the Walker A motif (*i*.*e*. PilT[K136] and PilU[K134]) and the atypical Walker B motif (*i*.*e*. PilT[E204] and PilU[E202]) are boxed in red. Species abbreviations: [**Vc**]; *Vibrio cholerae*, [**Pa**]; *Pseudomonas aeruginosa*, [**Nm**]; *Neisseria meningitidis*, [**Ng**]; *Neisseria gonorrhoeae*.

We first set out to establish baseline results in assays designed to test pilus function. In the case of the DNA-uptake pilus we have used chitin-independent competence induction to assay natural transformation and auto-aggregation, as these phenotypes provide reliable readouts on the state of pilus retraction [50, 52, 54]. Indeed, strains deleted for *pilT* exhibit a 1000-fold drop in transformation frequency and in liquid culture form large aggregates that rapidly sediment (Fig 1A and 1B). Under natural induction conditions on chitin surfaces, the effect of Δ*pilT* on transformation was even more prominent, with a 10,000-fold drop in transformation frequency, and equivalent to the frequencies observed with cells unable to make pili (S1 Fig). In contrast, deletion of *pilU* had no effect on transformation and did not promote aggregation (Fig 1A and 1B), in agreement with previous work showing that, under laboratory conditions at least, PilU is not required for DNA-uptake pilus function [50, 52].

To assay the functionality of the MSHA pilus we have used flagella-dependent swarming motility on soft agar. Indeed, Jones *et al*. previously demonstrated that the adhesive function of MSHA pili in surface attachment and near-surface motility restricts the ability of cells to swim on soft agar [47, 55]. Thus, cells unable to make MSHA pili or those with a loss of MSHA function exhibit an enhanced swarming motility phenotype [47]. As shown in Fig 1C our results recapitulate this phenotype, with strains lacking *mshA* or *pilT* exhibiting a clear gain of motility phenotype. Again, Δ*pilU* does not have an obvious effect on MSHA pilus function, as previously described [47]. Finally, in all cases, strains bearing a double Δ*pilTU* deletion behaved in an identical manner to those carrying the single Δ*pilT* deletion (Fig 1A–1C). Thus, these assays confirm that PilT is absolutely required for the normal function of both DNA-uptake pili and MSHA pili.

### The motility defect of Δ*pilT* is due to altered MSHA pilus biogenesis

**Fig 2:**
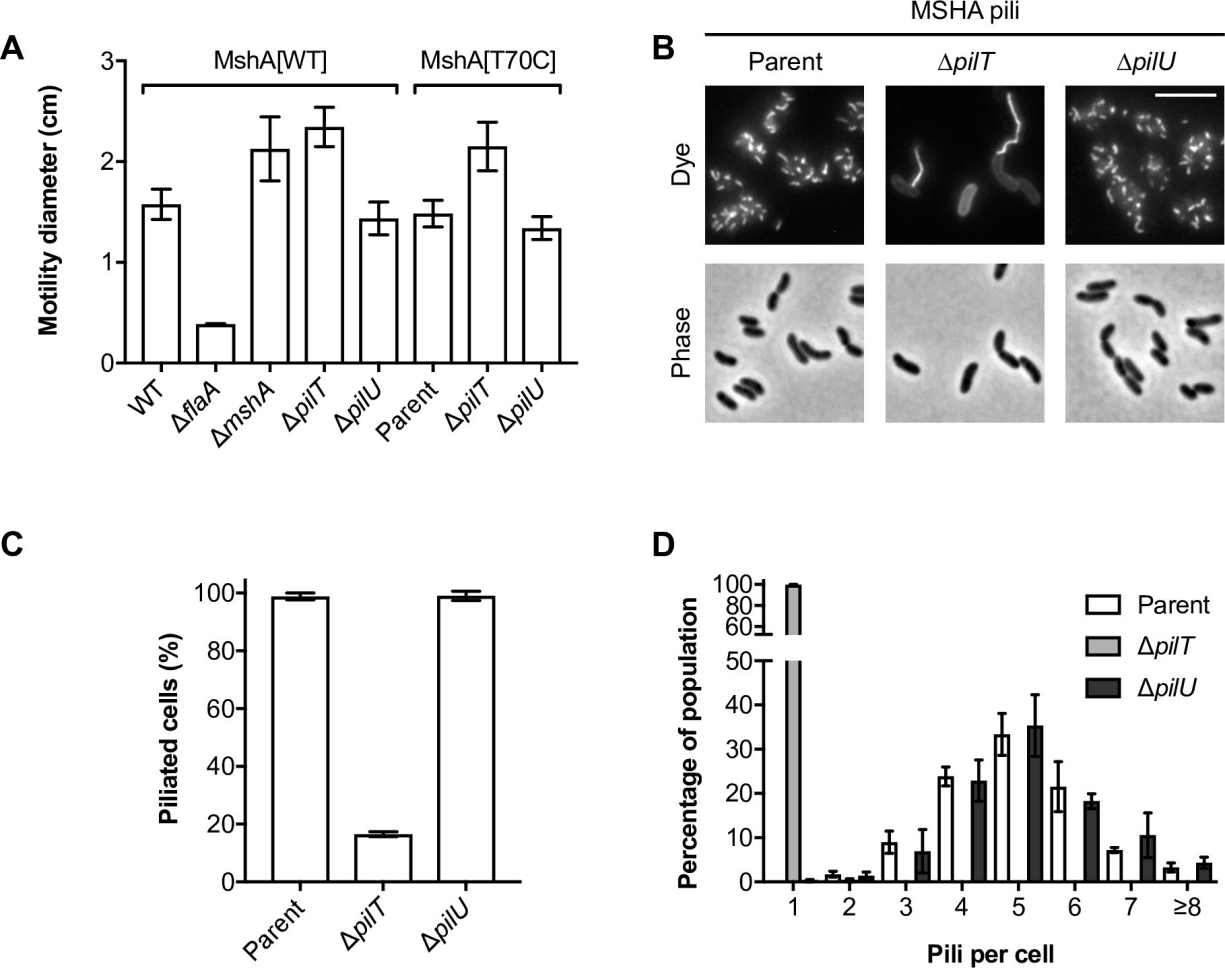
Loss of MSHA pili function in Δ*pilT* is due to altered pilus biogenesis. (**A**) Functionality of the MshA[T70C] cysteine variant and its derivatives was assessed by a motility assay alongside the relevant parental controls, as indicated. Surface motility was determined on soft LB agar plates. The swarming diameter (cm) is the mean of three repeats (±S.D.). A flagellin-deficient (Δ*flaA*) non-motile strain was used as a negative control. (**B**) Snapshot imaging of MSHA pili in WT parent (A1552-MshA[T70C]), Δ*pilT* (A1552-MshA[T70C], Δ*pilT*) and Δ*pilU* (A1552-MshA[T70C], Δ*pilU*) backgrounds, as indicated. Cells were stained with AF-488-Mal (Dye). Scale bar = 5 μm. (**C**-**D**) Quantification of MSHA piliation in snapshot imaging of WT parent, Δ*pilT* and Δ*pilU* backgrounds, as indicated. Bars represent the mean of three repeats (±S.D.). (**C**) Percentage of piliated cells. (**D**) Histogram of the number of pili per cell in piliated cells. *n* = *c*.*a*. 200–600 cells per strain per repeat.

By analogy to other T4aP systems, the loss of MSHA pilus function in Δ*pilT* cells has been presumed to result from the loss of pilus retraction. Indeed, in the initial description that PilT is required for MSHA pilus function, WT cells were reported as having single pili whereas Δ*pilT* cells had multiple pili, as might be expected [53]. However, Jones *et al*., recently reported that Δ*pilT* cells actually assembled less MSHA pili on the cell surface and did not observe hyper-piliation of Δ*pilT* cells by electron microscopy [47]. Since this is in contrast to what has been observed with other T4aP systems we therefore sought to confirm that PilT affects MSHA function directly. To do so, we used a previously described MshA cysteine variant (MshA[T70C]), which is the major pilin of the MSHA pilus, and that allows pilus visualisation by cysteine labelling [56, 57]. As shown in Fig 2A using the surface motility assay to test MSHA pilus function, the MshA[T70C] variant behaves in a manner indistinguishable from that of the parental controls.

Cells producing MshA[T70C] were near uniformly peritrichously piliated, with the majority of cells displaying 4–6 short (*c*.*a*. 0.5 μm) pili per cell (Fig 2B–2D). In agreement with the functional data above, the appearance of MSHA pili was not affected by the deletion of *pilU* (Fig 2B–2D). In sharp contrast, the piliation of Δ*pilT* cells dropped to less than 20%, with almost all of these cells displaying a single long pilus (Fig 2B–2D). Curiously, the length of these long pili (4.5 ± 0.51 μm; *n* = 281 pili) is approximately equivalent to the sum of those displayed on a WT cell. Thus, we speculate that these long pili might result from a ‘runaway’ extension event and that PilT might normally antagonise or otherwise cooperate with the extension ATPase MshE, to limit pilus length. In summary, these data show that PilT is required for proper MSHA pilus biogenesis, but that the MSHA pilus behaves in a manner distinct from that of the DNA-uptake pilus and other T4aP. Moreover, these data further support the idea that the enhanced motility phenotype of Δ*pilT* is due to the loss of MSHA pilus function [47] and reveal a useful additional readout for PilT function.

### Inactivating PilT reveals unexpected intermediate phenotypes that are PilU-dependent

ASCE ATPases such as PilT and PilU contain four characteristic and highly conserved motifs (S2 Fig) [13–16, 58]. The Walker A motif (WA; GX_4_GKS/T) is required for ATP-binding, whereas the atypical Walker B motif (WB; Dh_4_GE; h, hydrophobic) is required for ATP-hydrolysis and provides a catalytic glutamate to polarise an attacking water molecule (Fig 1D) [13, 14, 20, 21]. An Asp box containing acidic residues is involved in magnesium coordination and a His box containing a pair of histidines are both also required for function [13, 20, 21]. Thus, to investigate the role of ATPase activity in pilus retraction, we tested the functionality PilT and PilU variants bearing alanine substitutions in the invariant WA box lysine, predicted to disrupt ATP-binding, and in the invariant WB box glutamate, predicted to disrupt ATP-hydrolysis (Fig 1D).

**Fig 3:**
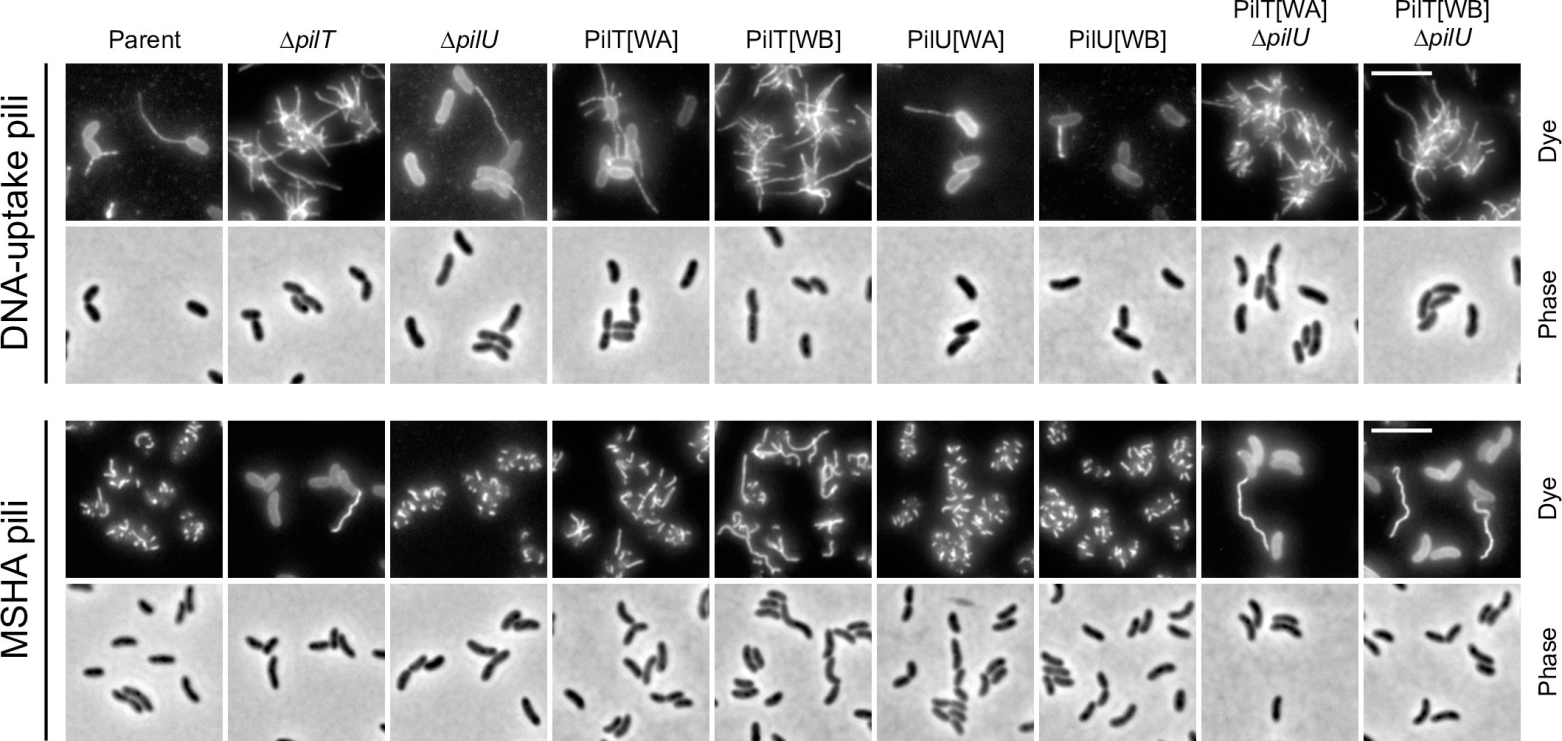
Cysteine labelling of DNA-uptake and MSHA pili in PilT and PilU variants. Visualisation of DNA-uptake pili (PilA[S67C]) and MSHA pili (MshA[T70C]) in the indicated backgrounds. To visualise DNA-uptake pili strains were cultured in the presence of chitin-independent competence induction. Cells were stained with AF-488-Mal (Dye). Scale bars = 5 μm.

As expected, PilU [WA] and [WB] variants did not affect either transformation, aggregation or motility (Fig 1A–1C). In contrast, the PilT [WA] and [WB] variants had only a modest transformation defect, as compared to Δ*pilT* (Fig 1A). This was unexpected, as both substitutions have previously been validated as abolishing ATPase activity *in vitro* and function *in vivo* [13, 14, 17, 59] in a variety of T4aP systems from other species. Indeed, the PilT[WA] variant did not promote auto-aggregation and showed normal motility (Fig 1B and 1C). Consistent with this, DNA-uptake pili and MSHA pili did not appear severely affected, although in the case of DNA-uptake pili we often observed cells with more than one pilus, and MSHA pili appeared to approximately double in length, but were otherwise unperturbed (Fig 3). In contrast, the PilT[WB] variant displayed additional and more severe phenotypes. Cells producing PilT[WB] were hyper-piliated for DNA-uptake pili and auto-aggregated, albeit at slightly reduced levels compared to Δ*pilT* (Fig 1B and Fig 3). Likewise, the PilT[WB] variant showed an enhanced motility phenotype similar to that of Δ*pilT* (Fig 1C). However, in contrast to the deletion, almost all cells displayed a mixture of long and short MSHA pili (Fig 3). Taken together these results indicate that substitutions designed to inactivate PilT produce intermediate phenotypes.

We hypothesised that partial functional redundancy of PilU could explain these unexpected intermediate phenotypes. If correct, then either deleting *pilU* or creating WA and WB variants of PilU in these backgrounds should lead to a total loss of pilus function. Indeed, cells producing PilT[WA] or [WB] variants that were co-deleted for *pilU* showed a total loss of DNA-uptake and MSHA pilus function and produced phenotypes equivalent to that of the Δ*pilT* mutant (Fig 1A–1C and Fig 3). Importantly, the combinations with PilU[WA] and [WB] variants also behaved similarly (Fig 1A–1C), indicating that the enduring pilus functionality that we observe in these backgrounds requires PilU ATPase activity. In summary, these data show that PilU can act as a *bona fide* retraction ATPase in *V*. *cholerae* and maintain the functionality of two distinct T4aP systems. Surprisingly, however, the results above suggest that PilU can only support pilus function in the presence of PilT-even when the latter is inactivated.

### PilU functions as a PilT-dependent retraction ATPase

**Fig 4:**
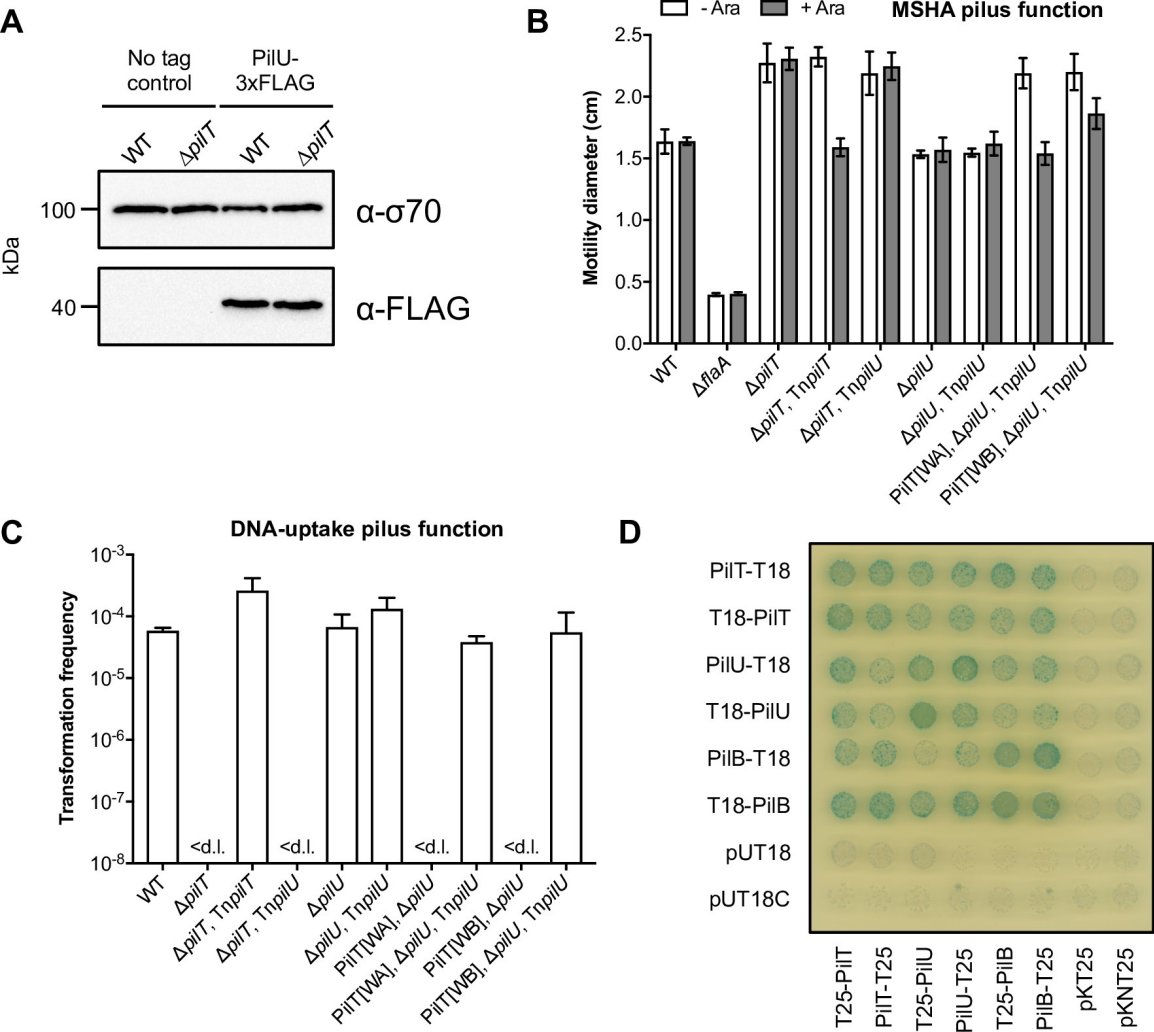
PilU functions as a PilT-dependent retraction ATPase. (**A**) Western blot of PilU-3xFLAG levels in cell lysates of strains encoding PilU-3xFLAG in a WT (A1552-PilU-3xFLAG) and Δ*pilT* (A1552-PilU-3xFLAG, Δ*pilT*) background, as indicated. Sample loading was verified using σ70 levels and the specificity of the anti-FLAG antibody was verified using the corresponding parental strain as a negative control. The predicted molecular mass of PilU-3xFLAG is 44 kDa. (**B**) Strains encoding arabinose-inducible versions of *pilT* (*araC P*_BAD_-*pilT*; Tn*pilT*) and *pilU* (*araC P*_BAD_-*pilU*; Tn*pilU*), within an ectopically integrated transposon, were tested for their ability to complement the enhanced motility phenotype of various backgrounds, in the absence (- Ara) and presence (+ Ara) of induction, as indicated. Surface motility was determined on soft LB agar plates. The swarming diameter (cm) is the mean of three repeats (±S.D.). A flagellin-deficient (Δ*flaA*) non-motile strain was used as a negative control. (**C**) Chitin-dependent transformation assay testing the ability of Tn*pilT* and Tn*pilU* to complement the transformation defect of various backgrounds. Transformation frequencies are the mean of three repeats (+S.D.). < d.l., below detection limit. All strains were cultured on chitin in the presence of arabinose. The corresponding parental strains without a transposon served as negative controls. (**D**) Interactions between the retraction ATPases PilT and PilU, and the extension ATPase PilB, were tested using a bacterial two-hybrid system. Representative image of *E*. *coli* strain BTH101 producing combinations of N and C-terminal fusions of PilT, PilU and PilB to the T18 and T25 domains of adenylate cyclase, as indicated. Empty vectors served as negative controls.

The data so far suggest that PilU is unable to function for pilus retraction in the absence of PilT. However, two distinct models could explain these observations. In the first model, PilU could actually be a separate retraction motor, but because *pilU* sits in an operon with *pilT*, the deletion of *pilT* might inadvertently disrupt PilU production. Indeed, certain *pilT* deletions have been reported to have such an effect on PilU production in *N*. *gonorrhoeae* and *P*. *aeruginosa* [41, 60]. In the second model, PilU would not form a separate retraction motor, but rather would function via a direct interaction with PilT. To distinguish between these possibilities, we first inserted a sequence coding for a 3xFLAG epitope tag at the 3’ end of *pilU* to create a PilU-3xFLAG fusion. Importantly, as shown in Fig 4A, a band corresponding to PilU-3xFLAG was readily detectable in otherwise WT cells and its levels were not noticeably affected by the deletion of *pilT*. Furthermore, using the same epitope tag approach we determined that PilT and PilU are both produced at similar levels (S3 Fig), indicating that the inability of PilU to function independently is not simply due to lower abundance relative to PilT.

Second, we created arabinose-inducible versions of *pilT* and *pilU* (*i*.*e*. Tn*pilT* and Tn*pilU*), which were integrated at an ectopic locus, and then tested for their ability to mediate pilus functionality in various backgrounds using swarming motility as a readout for MSHA pilus function and natural transformation on chitin as a readout for DNA-uptake pilus function. As shown in Fig 4B and 4C, ectopic production of PilT was sufficient to fully complement the enhanced motility phenotype and transformation defect of Δ*pilT*. Conversely, ectopic production of PilU showed no activity in the Δ*pilT* background (Fig 4B and 4C). As a control, to verify that Tn*pilU* was capable of generating PilU at levels sufficient to mediate pilus function, we re-tested it in backgrounds containing the inactivated PilT[WA] and [WB] variants. In contrast to the results above, ectopic production of PilU in these backgrounds was sufficient to fully complement both motility and transformation phenotypes (Fig 4B and 4C). Notably, PilU production also led to partial rescue of the PilT[WB] motility phenotype (Fig 4B), suggesting that it is likely being overproduced relative the WT situation. Indeed, Western blotting confirmed that Tn*pilU* induction leads to significant PilU overproduction (S4 Fig). Finally, previous work in *N*. *meningitidis* and *P*. *aeruginosa* has identified a network of interactions between the extension and retraction ATPases [61, 62]. As shown in Fig 4D, using the same bacterial two-hybrid assay approach, we also detected a similar interaction network between PilB, PilT and PilU. Thus, taken together, these results are consistent with the second model, in which PilU functions via a direct interaction with PilT.

The simplest interpretation of these results is that PilT is required to recruit PilU to the pilus machinery. If correct, then the inactivated PilT[WA] and [WB] variants would be expected to behave similarly. However, as detailed above, although both variants retain PilU-dependent function, the PilT[WB] variant has additional stronger defects than that of the [WA] variant. Given that ATP-binding and hydrolysis are linked to a series of conformational changes [20, 21, 25, 26], one explanation for this difference could be that the [WA], which is defective in ATP-binding, retains a greater degree of conformational freedom, as compared to the [WB] variant, which is unable to hydrolyse its bound ATP. Thus, these observations suggest that PilT does not simply recruit PilU but that they work together.

Given the ability of PilT and PilU to cross-interact, it has been hypothesised that they might intermix to form hetero-hexamers [35, 38, 61, 63]. However, our observation that the PilU[WA] and [WB] variants show no discernable phenotypes, even though we show above that they are indeed non-functional, argues against this idea. To test this more directly, we overproduced PilT and PilU and their respective [WA] and [WB] variants and assayed their ability to interfere with normal PilT function. As shown in S5 Fig PilT[WA] overproduction leads to dominant negative phenotypes in both MSHA pilus and DNA-uptake pilus function. In contrast, none of the PilU variants had an effect, supporting the idea that they do not intermix with PilT. Interestingly, but for reasons that we do not yet understand, the PilT[WB] variant also had no effect. In summary, we propose that PilU is not a separate retraction motor but that it exerts its function through PilT, and is therefore a PilT-dependent retraction ATPase.

Finally, previous work with domain swapped chimeras indicated that the N-terminal domains of PilT and PilU might be distinct [64]. Thus, to test if this could explain the PilT dependent behaviour of PilU we created similar chimeras in which the N- and C-terminal domains of PilT and PilU have been swapped *i*.*e*. PilT^N^-PilU^C^ and PilU^N^-PilT^C^ (S6A Fig). Strikingly, the PilU^N^-PilT^C^ chimera is able to support near-WT levels of transformation (S6B Fig), suggesting that the C-terminal domain of PilT likely contains specific residues required for mediating pilus function. However, this chimera was unable to support MSHA pilus function (S6C Fig) indicating that it is only partially functional and thus further work will be needed in this area.

### A naturally occurring PilT variant in pandemic *V*. *cholerae* MO10 renders PilU essential

**Fig 5:**
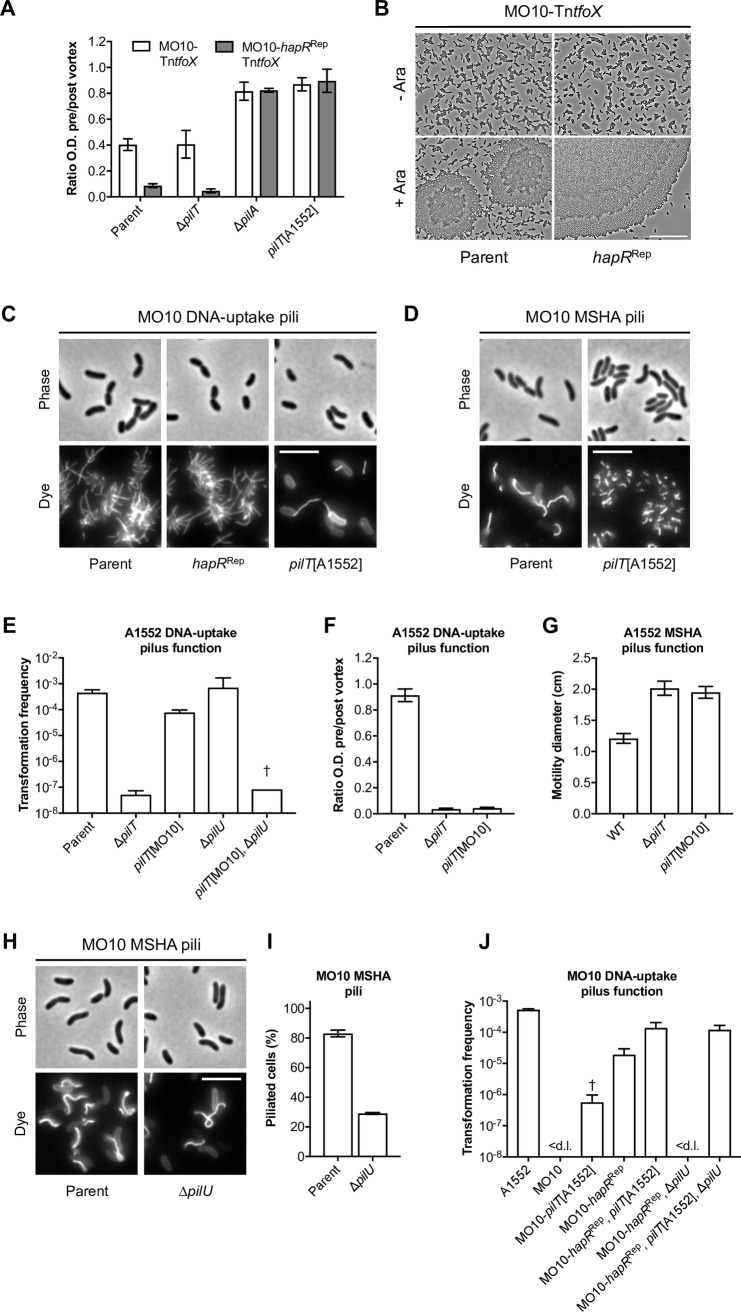
Pandemic *V*. *cholerae* strain MO10 contains a naturally occurring *pilT* mutation. (**A**) Derivatives of strain MO10 were tested for DNA-uptake pilus mediated aggregation in a MO10-Tn*tfoX* and a MO10-*hapR*^Rep^, Tn*tfoX* background, as indicated. Aggregation is shown as the ratio of the culture optical density (O.D. _600 nm_) before and after vortexing. Values close to 1 = No aggregation. Values close to 0 = Full aggregation. Strains were cultured in the presence of *tfoX* induction. Values are the mean of three repeats (±S.D.). (**B**) Phase-contrast microscopy of MO10 cells in the parental (MO10-Tn*tfoX*) and *hapR*^Rep^ (MO10-*hapR*^Rep^, Tn*tfoX*) backgrounds, grown in the absence (- Ara) and presence (+ Ara) of *tfoX* induction, as indicated. Scale bar = 25 μm. (**C**) Visualisation of DNA-uptake pili in the parental (MO10-Tn*tfoX*, PilA[S67C]), *hapR*^Rep^ (MO10-*hapR*^Rep^, Tn*tfoX*, PilA[S67C]) and *pilT*[A1552] (MO10-*hapR*^Rep^, Tn*tfoX*, PilA[S67C], *pilT*[A1552]) backgrounds, as indicated. Cells were cultured in the presence of *tfoX* induction and stained with AF-488-Mal (Dye). Scale bar = 5 μm. (**D**) Visualisation of MSHA pili in the parental (MO10-MshA[T70C]) and *pilT*[A1552] (MO10-MshA[T70C], *pilT*[A1552]) backgrounds, as indicated. Cells were stained with AF-488-Mal (Dye). Scale bar = 5 μm. (**E**-**G**) Functionality of MO10 PilT (*i*.*e*. *pilT*[MO10]) was assessed by transformation, aggregation and motility in strain A1552. (**E**) Chitin-independent transformation assay. Transformation frequencies are the mean of three repeats (+S.D.). †; below detection limit in two repeats. (**F**) Aggregation was done as in (A). (**G**) Surface motility assay on soft LB agar plates. The swarming diameter (cm) is the mean of three repeats (±S.D.). (**H**-**J**) MO10 requires PilU for pilus function. (**H**) Visualisation and (**I**) quantification of MSHA pili in the parental (MO10-MshA[T70C]) and Δ*pilU* (MO10-MshA[T70C], Δ*pilU*) backgrounds, as indicated. Cells were stained with AF-488-Mal (Dye). Scale bar = 5 μm. *n* = 400–600 cells counted per strain per repeat. (**J**) Chitin-independent transformation assay. Transformation frequencies are the mean of three repeats (+S.D.). †; below detection limit in one repeat.

We previously showed that representative 7^th^ pandemic O1 El Tor strains are all equally capable of auto-aggregation via their DNA-uptake pili, but that in liquid culture this is manifest only when *pilT* is deleted [52]. To extend this work we have now also investigated strain MO10, which is often used as a representative of the O139 serogroup subtype. In 1992 strains belonging to this serogroup caused a severe cholera epidemic that spread rapidly throughout the Indian subcontinent and, to date, remains the only serogroup other than O1 known to cause pandemic disease [65–69]. Unexpectedly, competence-induced cells of MO10 aggregated in an otherwise unmodified background (Fig 5A and 5B). Indeed, deletion of *pilT* did not affect the level of aggregation (Fig 5A). Since MO10 carries a defective variant of the quorum-sensing regulator HapR [70], and transformation is quorum-sensing dependent, we first repaired it to that of the canonical A1552 HapR (= *hapR*^Rep^). Nonetheless, this phenotype was not dependent on the defective HapR variant since the aggregation phenotype was further enhanced when it was repaired (Fig 5A and 5B). However, in agreement with our previous work, deletion of *pilA* abolished aggregation (Fig 5A). Thus, these results suggest that MO10 has a defect in DNA-uptake pilus retraction.

Inspection of the MO10 *pilTU* locus revealed a single bp mutation in *pilT*, resulting in the substitution of a normally invariant arginine (R206S) that sits directly adjacent to the Walker B motif (S2 and S7 Figs). R206 forms part of a network of arginine residues that surround the ATP-binding site and that are predicted to be required for proper PilT function [20]. Indeed, compared to the situation in A1552 (Fig 3), competence-induced cells of MO10 were hyper-piliated for DNA-uptake pili, irrespective of the *hapR* repair (Fig 5C). Likewise, MSHA pilus biogenesis was also clearly altered (Fig 5D), with the majority of cells having one or more long pili. In both cases, the effects on pili appear similar to the PilT[WB] variant examined above. Taken together, these data suggest that MO10 PilT is defective. The evidence for this assertion is as follows. First, replacing *pilT*[MO10] with that of A1552 abolished the ability of MO10 to aggregate (Fig 5A) and restored the expected configuration of DNA-uptake and MSHA pili (Fig 5C and 5D). Second, recreating the *pilT*[MO10] mutation in A1552 reduced transformation frequency, allowed competence-induced cells to aggregate, and produced an enhanced motility phenotype (Fig 5E–5G). Third, similar to the PilT[WB] variant described above, natural transformation of cells carrying *pilT*[MO10] was rendered PilU-dependent (Fig 5E). Finally, deleting *pilU* in strain MO10 led to a dramatic drop in MSHA pilus biogenesis, such that now only a subpopulation of cells had pili (Fig 5H and 5I), similar to that observed above for retraction-deficient A1552 (Fig 2B and 2C). Moreover, deleting *pilU* also abolished transformation, unless the MO10 PilT variant was first replaced with that of A1552 (Fig 5J).

In summary, MO10 carries a naturally occurring defective PilT variant, which affects multiple processes, and requires PilU to maintain pilus function. Importantly, although multiple O139 genomes have now been sequenced, the MO10 PilT sequence is unique. Moreover, with respect to this variant, the genomes of four contemporary O139 isolates all contain a canonical PilT identical to that of A1552 [71]. Consequently, our data suggest that caution should be used when interpreting pilus related phenotypes in MO10 (*e*.*g*. surface colonisation via MSHA and subsequent biofilm formation), as this strain may not be a representative isolate.

### *P*. *aeruginosa* PilU also functions in a PilT-dependent manner

**Fig 6:**
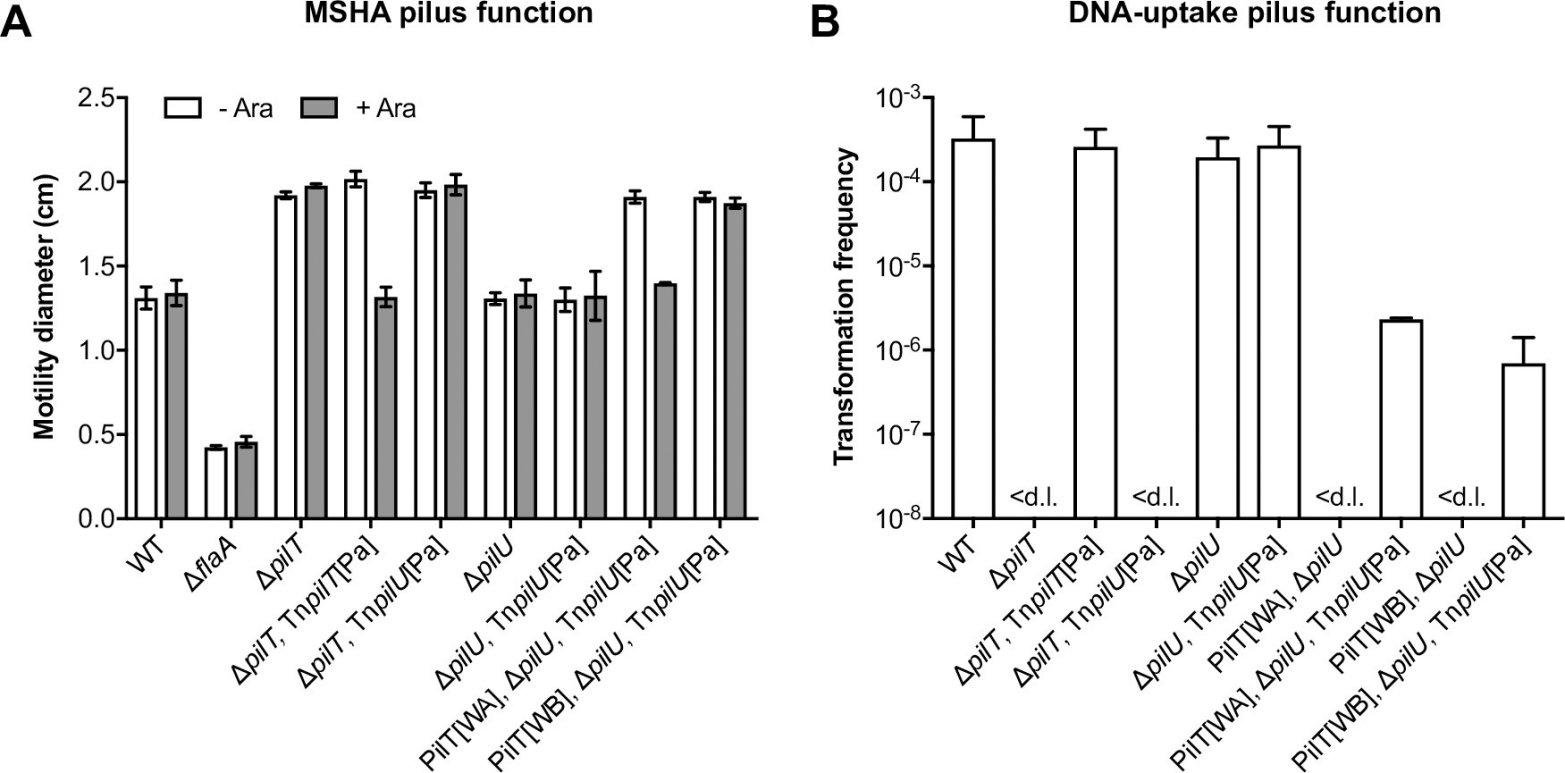
*P*. *aeruginosa* PilU functions in a PilT-dependent manner. (**A**) Strains encoding arabinose-inducible versions of *P*. *aeruginosa pilT* (*araC P*_BAD_-*pilT*[Pa]; Tn*pilT*[Pa]) and *pilU* (*araC P*_BAD_-*pilU*[Pa]; Tn*pilU*[Pa]), within an ectopically integrated transposon, were tested for their ability to complement the enhanced motility phenotype of various backgrounds, in the absence (- Ara) and presence (+ Ara) of induction, as indicated. Surface motility was determined on soft LB agar plates. The swarming diameter (cm) is the mean of three repeats (±S.D.). A flagellin-deficient (Δ*flaA*) non-motile strain was used as a negative control. (**B**) Chitin-dependent transformation assay testing the ability of Tn*pilT*[Pa] and Tn*pilU*[Pa] to complement the transformation defect of various backgrounds. Transformation frequencies are the mean of three repeats (+S.D.). < d.l., below detection limit. All strains were cultured on chitin in the presence of arabinose. The corresponding parental strains without a transposon served as negative controls.

To test if the functional coupling between PilU and PilT is conserved, we examined the ability of *P*. *aeruginosa* PilT and PilU to function in *V*. *cholerae*. To do so, we again used ectopically integrated arabinose-inducible constructs, *i*.*e*. Tn*pilT*[Pa] and Tn*pilU*[Pa], and tested their respective abilities to support the functionality of the MSHA pilus during surface motility and the DNA-uptake pilus during natural transformation on chitin. As shown in Fig 6A and 6B, the production of PilT[Pa] was sufficient to completely counteract both the enhanced motility phenotype and the transformation defect that are present in a Δ*pilT* background. In contrast, PilU[Pa] showed no such activity in a Δ*pilT* background (Fig 6A and 6B). Strikingly, however, PilU[Pa] production was sufficient to fully counteract the enhanced motility phenotype of the PilT[WA] Δ*pilU* background (Fig 6A). Likewise, PilU[Pa] also showed the ability to restore natural transformation on chitin in both inactivated PilT backgrounds (Fig 6B), albeit at reduced levels compared to that of PilU[Vc]. In summary, these data demonstrate that PilT[Pa] and PilU[Pa] can support the functions of the MSHA and DNA-uptake pili in *V*. *cholerae*. Moreover, these data indicate that although PilT[Pa] can function as an independent retraction ATPase, PilU[Pa] does not, and therefore provide evidence that PilU[Pa], like PilU[Vc], also functions as a PilT-dependent retraction ATPase.

### Concluding remarks

Here we demonstrate that when PilT is inactivated, via substitutions in the Walker A and B boxes, the PilT paralogue PilU can maintain the near normal functionality of two distinct T4aP systems, namely the DNA-uptake pilus and the MSHA pilus. We show that the ability of PilU to function as a retraction motor is dependent on the presence of functional Walker A and B boxes. Moreover, we establish that PilU is unable to function in the absence of PilT. Previous work in a wide range of bacteria had concluded that PilU does not play a major role in retraction; based mainly on the observation that Δ*pilT* leads to a total loss of function [29, 31, 33, 35–38, 40, 41, 60]. Additionally, some groups had noticed that PilU was unable to complement Δ*pilT*, leading Brown *et al*., to suggest that PilU might not be a separate retraction motor [31, 35, 40]. The demonstration here that PilU acts as a PilT-dependent retraction motor provides direct evidence for this idea and likely explains these initially puzzling observations. Indeed, given our discovery that *P*. *aeruginosa* PilU also acts in a PilT-dependent manner, our findings in *V*. *cholerae* likely extend to other bacteria, and suggest that the functional coupling between PilU and PilT could be a widespread mechanism for optimising pilus retraction.

While this work was under completion [72], a recent preprint by Chlebek *et al*., has also examined PilU function and reached similar conclusions to those reported here [73]. Notably, these authors went on to show that despite the lack of a functional phenotype, the deletion of *pilU* reduces both the rate and force of DNA-uptake pilus retraction. Moreover, Chlebek *et al*., also showed that PilU functions in a similar PilT-dependent manner in *A*. *baylyii*. This, taken together with our demonstration that *P*. *aeruginosa* PilU also functions in a PilT-dependent manner, adds to the idea that this is likely a conserved feature of PilU. Interestingly, *Neisseria* sp. carry an additional PilT paralogue, PilT-2, which in *N*. *gonorrhoeae* was shown by Kurre *et al*., to modulate T4aP retraction speed and enhance twitching motility [38]. Moreover, it was shown that in *N*. *meningitidis pilT-2*, like *pilU*, was unable to cross-complement a Δ*pilT* strain [35]. Thus, an interesting hypothesis to test in future work is that PilT-2 also functions in a PilT-dependent manner.

Compared to PilT, the requirement for PilU function appears to be context dependent. For example in *P*. *aeruginosa*, PilU is only required for twitching motility underneath agar, which represents a high-friction environment [74]. Similar specialised functions have been reported in other bacteria [31, 33, 35, 37, 40–42]. The functional coupling between PilU and PilT likely facilitates this versatility by ensuring that the action of the second motor is always coordinated with that of the first, and thus allows the system to deal with a range of loads without the need for separate retraction motors of varying strengths that could interfere with one another. An outstanding question that remains unclear is when PilU acts during the lifecycle of *V*. *cholerae*. Our data demonstrate that in strain MO10, PilU is required to maintain pilus functionality due a naturally occurring defect in PilT. However, acting as a ‘backup’ in this way is unlikely to be a primary role. Indeed, PilU is more likely required under certain environmental scenarios that require increased power *e*.*g*. to retract pili bound either to very large fragments of DNA or within the complex gel-like environments found in biofilms. PilU may also be required to power pilus retraction upon surface attachment. Finally, a key question going forward will be to determine how PilU directs its function via PilT. In the simplest model PilU would form an independent hexamer that sits directly beneath PilT. Curiously, PilT contains a highly conserved C-terminal motif (AIRNLIRE; S2 Fig) that is absent in PilU [59]. One possibility is that this motif is required either for the interaction with, or else the functional coupling with PilU. In support of this idea, the proposed interaction of the PilT hexamer with PilC, which forms the base of the pilus machinery, orients the protein such that the AIRNLIRE motif faces the cytoplasm [25, 26].

## Materials and methods

### Bacterial strains and plasmids

The bacterial strains and plasmids used in this study are listed in S1 Table. The *V*. *cholerae* strain used throughout this work, A1552 [75], is a fully-sequenced [76] toxigenic O1 El Tor Inaba strain representative of the on-going 7^th^ cholera pandemic.

### General methods

Bacterial cultures were grown aerobically at 30˚C or 37˚C, as required. Liquid medium used for growing bacterial strains was Lysogeny Broth (LB-Miller; 10 g/L NaCl, Carl Roth, Switzerland) and solid medium was LB agar. Ampicillin (Amp; 100 μg/mL), gentamicin (Gent; 50 μg/mL), kanamycin (Kan; 75 μg/mL), streptomycin (Str; 100 μg/mL) and rifampicin (Rif; 100 μg/mL) were used for selection, as required. To induce expression from the *P*_BAD_ promoter, cultures were grown in media supplemented with 0.2% L-arabinose. Natural transformation of *V*. *cholerae* on chitin flakes was done in 0.5x DASW (defined artificial seawater), supplemented with vitamins (MEM, Gibco) and 50 mM HEPES, as previously described [49]. Thiosulfate citrate bile salts sucrose (TCBS; Sigma-Aldrich, Switzerland) agar was used to counter-select for *E*. *coli* following bacterial mating. SacB-based counter-selection was done on NaCl-free medium containing 10% sucrose.

### Strain construction

Molecular cloning was performed using standard methods [77]. All constructs were verified by PCR and Sanger sequencing (Microsynth AG, Switzerland). Genetic engineering of *V*. *cholerae* was done using either a combination of natural transformation and FLP-recombination (TransFLP; [78–80]) or allelic exchange using bi-parental mating and the counter-selectable plasmid pGP704-Sac28 [46]. Tri-parental mating was used to integrate the mini-Tn7 transposon carrying *araC* and various *P*_BAD_-driven genes into the large chromosome, as previously described [81].

### Chitin-independent competence induction

Natural competence was induced in liquid culture using an established chitin-independent approach that relies on the integration of a mini-Tn7 transposon containing an arabinose-inducible copy of *tfoX* (*i*.*e*. *araC*, *P*_BAD_-*tfoX*), which we refer to as Tn*tfoX*. [54]. In the presence of inducer, strains carrying Tn*tfoX* turn on the expression of the competence genes according to the known regulatory pathways and upon reaching high cell-density are transformable at levels similar to those seen on chitin [54]. In the absence of inducer strains are non-transformable [54].

### Chitin-independent transformation assay

A chitin-independent transformation assay was used to assess transformation in strains carrying Tn*tfoX*, as previously described [50, 54]. Briefly, overnight cultures were back-diluted 1:100 and grown 3h at 30˚C with shaking (180 rpm). Genomic DNA (derived from strain A1552-lacZ-Kan) was added to a final concentration of 2 μg/mL and cultures incubated for 5h at 30˚C with shaking (180 rpm). Transformation frequency was calculated as the number of kanamycin resistant transformants divided by the total number of bacteria.

### Chitin-dependent transformation assay

Natural transformation, without and with enrichment, was done as previously described [50]. Otherwise, natural transformation on chitin was performed as previously described, with slight modifications [79]. Chitin flakes were submerged within 950 μL 0.5x DASW + HEPES + Vitamins in a 1.5 mL eppendorf tube. 50 μL of overnight culture was added, vortexed briefly to mix and incubated standing for 8h at 30˚C, at which point arabinose was added (final 0.2%) to induce the expression of the various transposon-encoded *pilT* and *pilU*. 24h after the initial inoculation, 2 μg genomic DNA (derived from strain A1552-lacZ-Kan) was added, mixed by inversion, and incubated at 30˚C for 7h. Bacteria were detached from chitin flakes by vortexing, before serial dilution and enumeration, as described above.

### Aggregation assay

DNA-uptake pilus mediated aggregation was quantified as previously described [52]. Briefly, overnight cultures were back-diluted 1:100 in LB + 0.2% arabinose and grown at 30˚C for 6h in 14 mL round bottom polystyrene test tubes (Falcon, Corning) on a carousel-style rotary wheel (40 rpm). Aggregates were allowed to settle by standing the tube at RT for 30 min. Aggregation was determined by measuring the optical density at 600 nm (O.D._600_) before and after mechanical disruption (vortex max speed; ~5 sec), which serves to disperse any settled aggregates, and is expressed as the ratio of the O.D._600_ Pre/Post-vortexing.

### Motility assay

Motility phenotypes were quantified by spotting 2 μL of an overnight culture onto soft LB agar (0.3%) plates (two technical replicates). Plates were incubated at RT for 24h prior to photography. The swarming diameter (cm) is expressed as the mean of three independent biological repeats. A flagellin-deficient (Δ*flaA*) non-motile strain served as a negative control.

### Microscopy

Cells were mounted on microscope slides coated with a thin agarose pad (1.2% w/v in PBS), observed using a Zeiss Axio Imager M2 epi-fluorescence microscope, and images analysed and prepared for publication using ImageJ (http://rsb.info.nih.gov/ij), as previously described [52].

### Cysteine labelling of DNA-uptake pili and MSHA pili

Overnight cultures were back-diluted 1:100 and grown at 30˚C for 3.5-4h on a rotary wheel, as above, in the absence (MSHA pili) and presence (DNA-uptake pili) of competence induction, as required. Pilus labelling was performed as previously described [52, 56]. 100 μL of culture was mixed with AF-488-Mal (Alexa Fluor 488 C_5_ Maleimide; Thermo Fisher Scientific; Cat# A10254) at a final concentration of 25 μg/mL and incubated at RT for 5 min in the dark. Labelled cells were harvested by centrifugation (5000 x *g*; 1 min), washed once with LB, re-suspended in 200 μL LB and imaged immediately.

### Western blotting

Overnight cultures were back-diluted 1:100 and grown in LB at 30˚C for 6h. Lysates were prepared by suspending harvested cells in an appropriate volume of 2x Laemmli buffer (100 μL buffer per O.D. unit) and boiling at 95˚C for 15 min. Proteins were resolved by SDS-PAGE, blotted onto PVDF membranes using a wet-transfer apparatus and immuno-detection was performed as described previously [54]. Primary anti-FLAG antibodies (Monoclonal ANTI-FLAG M2, Sigma; Cat# F1804) were used at a dilution of 1:2000. Anti-Mouse IgG HRP (Sigma; Cat# A5278) diluted 1:5000 was used as a secondary antibody. Sample loading was verified with Direct-Blot HRP anti-*E*. *coli* RNA Sigma70 (BioLegend; Cat# 663205) diluted 1:10,000.

### Bacterial two-hybrid assay

To study potential interactions between T4aP ATPases a Bacterial Adenylate Cyclase-Based Two-Hybrid (BACTH) system was employed (Euromedex, Cat# EUK001) [82]. PCR fragments carrying *pilB*, *pilT* and *pilU* were cloned into each of the BACTH vectors, which were routinely maintained in *E*. *coli* XL-10 Gold. Each vector pair was then introduced into chemically competent cells of *E*. *coli* BTH101, in all possible combinations. Empty vectors were used as negative controls. 5 μL of each transformation reaction was spotted onto LB plates containing 50 μg/mL Kan, 100 μg/mL Amp, 0.5 mM IPTG (isopropyl-β-D-thiogalactopyranoside), 40 μg/mL X-gal (5-Bromo-4-chloro-3-indolyl-β-D-galactopyranoside) and incubated at 30˚C for 40h prior to photography.

### Data reproducibility

All data are representative of the results of three independent biological repeats. All replication attempts were successful. Bar graphs show the mean value, error bars specify the standard deviation.

## Supporting information

S1 Fig Controls for chitin-dependent transformation assay. Strains deleted for either *pilA*, *pilB* or *pilT* all display a similar defect in natural transformation, with frequencies at or just above the detection limit. Transformation frequencies are the mean of three repeats (+S.D.). < d.l., below detection limit. #; < d.l. in 1 experiment, †; < d.l. in 2 experiments. A1552Δ*comEA* served as a negative control. Chitin-induced natural transformation and enrichment was performed as previously described (see methods). Briefly, to enrich cultures prior to plating, bacteria were detached from the chitin surfaces, transferred to 2-YT broth (2x Yeast extract and Tryptone) and cultured for 7h at 30˚C before plating and subsequent enumeration. The average detection limit in the absence of enrichment was 5.9 x 10^−9^ ± 7.8 x 10^−10^, and in the presence of enrichment was 2.1 x 10^−9^ ± 7.7 x 10^−11^.
(PDF)

S2 Fig Species-wide alignment of PilT and PilU. PilT and PilU protein sequences were aligned using Clustal Omega and the figure prepared using Jalview. Residues are shaded in graduations of blue according to sequence identity. Regions corresponding to the conserved Walker A box, Asp box, Walker B box and His box are highlighted in red. The conserved AIRNLIRE motif in PilT is also highlighted. A dashed red box indicates the residue (R206) that is substituted in *V*. *cholerae* strain MO10. Species abbreviations: [**Vc**]; *Vibrio cholerae* (CP028894.1), [**Pa**]; *Pseudomonas aeruginosa* (NC_002516.2), [**Ps**]; *Pseudomonas stutzeri* (CAB56295.1 and CAB56296.1), [**Nm**]; *Neisseria meningitidis* (FM999788.1), [**Ng**]; *Neisseria gonorrhoeae* (NC_002946.2), [**Dn**]; *Dichelobacter nodosus* (CP000513.1), [**Ab**]; *Acinetobacter baylyi* (NC_005966.1), [**Aa**]; *Aquifex aeolicus* (NC_000918.1), [**Mx**]; *Myxococcus xanthus* (NC_008095.1). Note that for simplicity, because *M*. *xanthus* has four additional PilT paralogues, only the experimentally validated PilT was included.
(PDF)

S3 Fig PilT and PilU are produced at similar levels. Western blot comparing PilT-3xFLAG and PilU-3xFLAG levels in cell lysates of strains A1552-PilT-3xFLAG and A1552-PilU-3xFLAG, as indicated. Sample loading was verified using σ70 levels and the specificity of the anti-FLAG antibody was verified using the cell lysate of the parental A1552 WT strain as a negative control. The predicted molecular mass of PilT-3xFLAG is 40.9 kDa and of PilU-3xFLAG is 44 kDa.
(PDF)

S4 Fig Tn*pilU* induction results in PilU overproduction. Western blot comparing PilU-3xFLAG levels in cell lysates of strains encoding PilU-3xFLAG either at its native locus (A1552-PilU-3xFLAG) or produced from an ectopically integrated transposon carrying an arabinose-inducible *araC P*_BAD_-*pilU-*3xFLAG construct (A1552-Tn*pilU*-3xFLAG). Cultures were grown in the absence (- Ara) and presence (+ Ara) of inducer, as indicated. Sample loading was verified using σ70 levels and the specificity of the anti-FLAG antibody was verified using the cell lysate of the parental A1552 WT strain as a negative control. The predicted molecular mass of PilU-3xFLAG is 44 kDa.
(PDF)

S5 Fig Ectopic production of PilT[WA] but not PilU[WA] is dominant negative. (**A**-**B**) Strains encoding various arabinose-inducible variants of *pilT* (*araC P*_BAD_-*pilT*; Tn*pilT*) and *pilU* (*araC P*_BAD_-*pilU*; Tn*pilU*), within an ectopically integrated transposon, were tested for their ability to interfere with normal PilT function using (**A**) surface motility as a readout for MSHA pilus function and (**B**) natural transformation as a readout for DNA-uptake pilus function. To avoid interference, all variants were tested in a Δ*pilU* background. The corresponding parental strains without a transposon served as negative controls. (**A**) Surface motility was determined on soft LB agar plates, in the absence (- Ara) and presence (+ Ara) of induction, as indicated. The swarming diameter (cm) is the mean of three repeats (±S.D.). The gain of motility phenotype of the A1552Δ*pilT* served as a positive control. (**B**) Chitin-dependent transformation assay. Transformation frequencies are the mean of three repeats (+S.D.). < d.l., below detection limit. All strains were cultured on chitin in the presence of arabinose. The loss of transformation phenotype of the A1552Δ*pilT* served as a positive control.
(PDF)

S6 Fig A domain swapped PilU-PilT chimera is partially functional. (**A**-**C**) Strains encoding arabinose-inducible chimeras, within an ectopically integrated transposon, in which the N-terminal (NTD) and C-terminal (CTD) domains of PilT and PilU have been swapped *i*.*e*. PilT^N^-PilU^C^ (*araC P*_BAD_-*pilT*^N^-*pilU*^C^; Tn*pilT*^N^-*pilU*^C^) and PilU^N^-PilT^C^ (*araC P*_BAD_-*pilU*^N^-*pilT*^C^; Tn*pilU*^N^-*pilT*^C^), were tested for functionality using (**B**) natural transformation as a readout for DNA-uptake pilus function and (**C**) surface motility as a readout for MSHA pilus function. To avoid interference, all variants were tested in a Δ*pilTU* background. The corresponding parental strains without a transposon served as negative controls. (**A**) The schematic illustrates the construction of the domain swapped PilT-PilU chimeras. The numbers in parentheses denote the source amino acid numbers of each domain. (**B**) Chitin-dependent transformation assay. Transformation frequencies are the mean of three repeats (+S.D.). < d.l., below detection limit. All strains were cultured on chitin in the presence of arabinose. The ability of Tn*pilT* to complement the transformation phenotype of A1552Δ*pilTU* served as a positive control. (**C**) Surface motility was determined on soft LB agar plates, in the absence (- Ara) and presence (+ Ara) of induction, as indicated. The swarming diameter (cm) is the mean of three repeats (±S.D.). The ability of Tn*pilT* to complement the gain of motility phenotype of A1552Δ*pilTU* served as a positive control.
(PDF)

S7 Fig Comparison of PilT and PilU from *V. cholerae* strains A1552 and MO10. (**A**-**C**) PilT and PilU protein sequences from *V*. *cholerae* strains A1552 and MO10 were aligned using Clustal Omega and the figure prepared using Jalview. MO10 PilT and PilU sequences were identified using the MO10 genome sequence, GCA_000152425.1. (**A**) Alignments highlighting the conserved Walker A and Walker B motifs of PilT and PilU from strains A1552 and MO10. Residues are shaded in graduations of blue according to sequence identity. The R206S substitution present in PilT[MO10] is boxed in red. (**B**-**C**) Full-length alignments demonstrating that (**B**) PilT and (**C**) PilU are otherwise identical.
(PDF)

S1 Table Bacterial strains and plasmids used in this study. (DOCX)
