# Supplementary figures and images for: The type IV pilus protein PilU functions as a PilT-dependent retraction ATPase
Source: PLoS Genet. 2019 Sep 16;15(9):e1008393. doi: 10.1371/journal.pgen.1008393 (PMC6762196; doi:10.1371/journal.pgen.1008393)

Transformation frequency

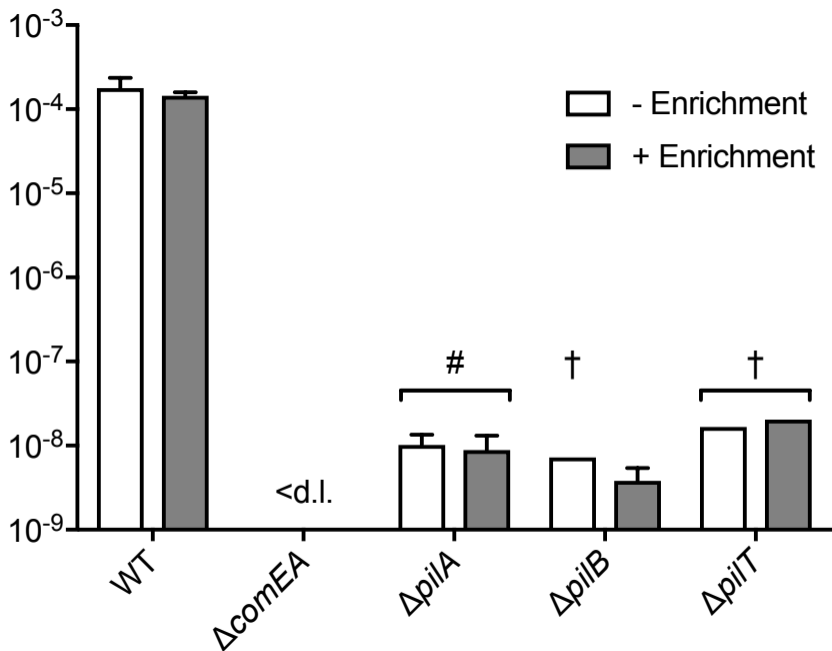

Supplement: S1 Fig — Strains deleted for either pilA, pilB or pilT all display a similar defect in natural transformation, with frequencies at or just above the detection limit. Transformation frequencies are the mean of three repeats (+S.D.). < d.l., below detection limit. #; < d.l. in 1 experiment, †; < d.l. in 2 experiments. A1552ΔcomEA served as a negative control. Chitin-induced natural transformation and enrichment was performed as previously described (see methods). Briefly, to enrich cultures prior to plating, bacteria were detached from the chitin surfaces, transferred to 2-YT broth (2x Yeast extract and Tryptone) and cultured for 7h at 30˚C before plating and subsequent enumeration. The average detection limit in the absence of enrichment was 5.9 x 10^−9^ ± 7.8 x 10^−10^, and in the presence of enrichment was 2.1 x 10^−9^ ± 7.7 x 10^−11^. (PDF) [file pgen.1008393.s001.pdf]

kDa

100

WT

*pilT*-3xFLAG

*pilU*-3xFLAG

$\alpha$ - $\sigma$ 70

40

$\alpha$ -FLAG

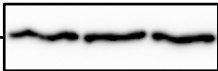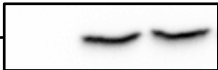

Supplement: S3 Fig — Western blot comparing PilT-3xFLAG and PilU-3xFLAG levels in cell lysates of strains A1552-PilT-3xFLAG and A1552-PilU-3xFLAG, as indicated. Sample loading was verified using σ70 levels and the specificity of the anti-FLAG antibody was verified using the cell lysate of the parental A1552 WT strain as a negative control. The predicted molecular mass of PilT-3xFLAG is 40.9 kDa and of PilU-3xFLAG is 44 kDa. (PDF) [file pgen.1008393.s003.pdf]

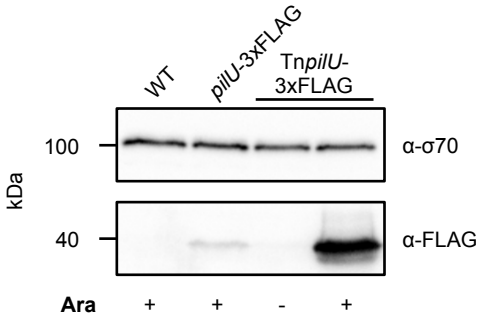

Supplement: S4 Fig — Western blot comparing PilU-3xFLAG levels in cell lysates of strains encoding PilU-3xFLAG either at its native locus (A1552-PilU-3xFLAG) or produced from an ectopically integrated transposon carrying an arabinose-inducible araC PBAD-pilU-3xFLAG construct (A1552-TnpilU-3xFLAG). Cultures were grown in the absence (- Ara) and presence (+ Ara) of inducer, as indicated. Sample loading was verified using σ70 levels and the specificity of the anti-FLAG antibody was verified using the cell lysate of the parental A1552 WT strain as a negative control. The predicted molecular mass of PilU-3xFLAG is 44 kDa. (PDF) [file pgen.1008393.s004.pdf]

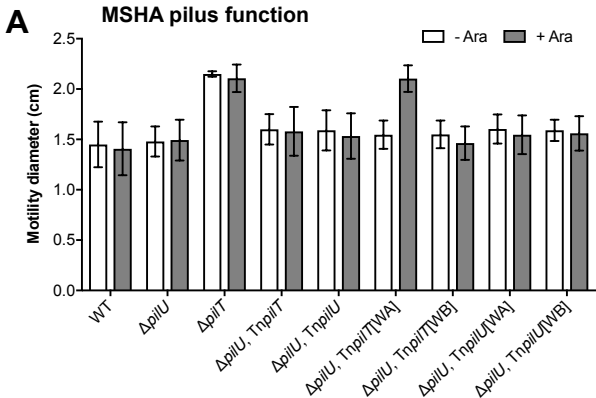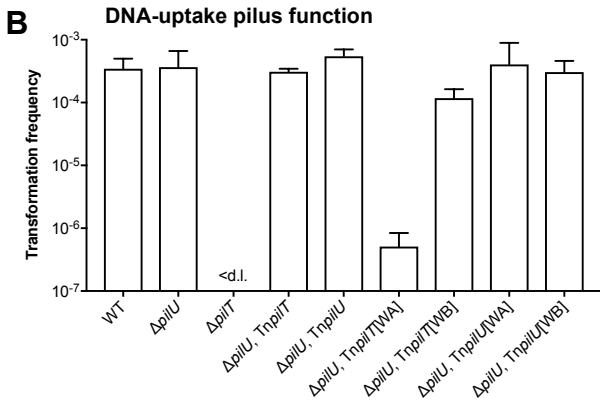

Supplement: S5 Fig — (A-B) Strains encoding various arabinose-inducible variants of pilT (araC PBAD-pilT; TnpilT) and pilU (araC PBAD-pilU; TnpilU), within an ectopically integrated transposon, were tested for their ability to interfere with normal PilT function using (A) surface motility as a readout for MSHA pilus function and (B) natural transformation as a readout for DNA-uptake pilus function. To avoid interference, all variants were tested in a ΔpilU background. The corresponding parental strains without a transposon served as negative controls. (A) Surface motility was determined on soft LB agar plates, in the absence (- Ara) and presence (+ Ara) of induction, as indicated. The swarming diameter (cm) is the mean of three repeats (±S.D.). The gain of motility phenotype of the A1552ΔpilT served as a positive control. (B) Chitin-dependent transformation assay. Transformation frequencies are the mean of three repeats (+S.D.). < d.l., below detection limit. All strains were cultured on chitin in the presence of arabinose. The loss of transformation phenotype of the A1552ΔpilT served as a positive control. (PDF) [file pgen.1008393.s005.pdf]

**A**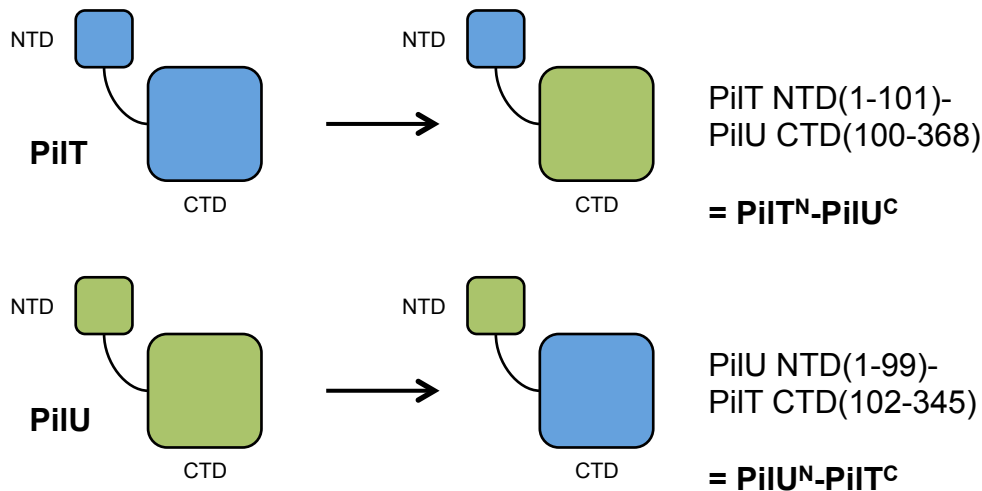**B****DNA-uptake pilus function**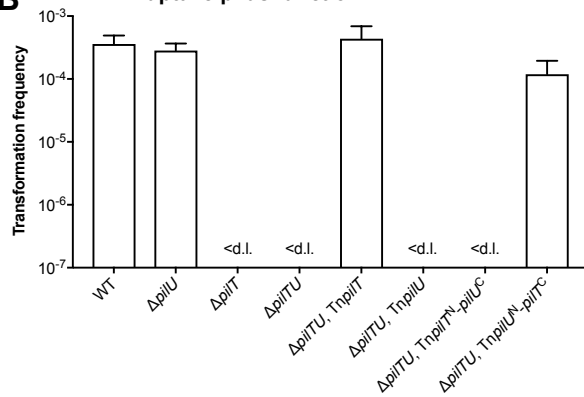**C****MSHA pilus function**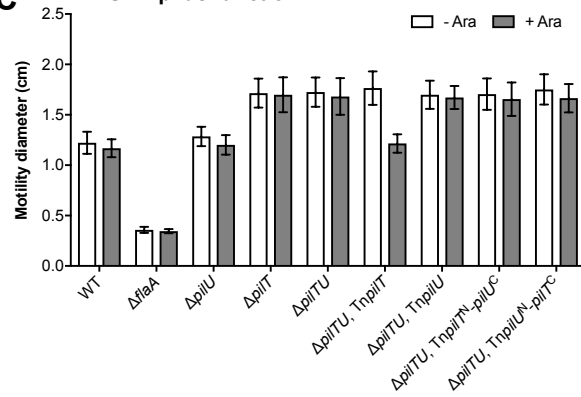

Supplement: S6 Fig — (A-C) Strains encoding arabinose-inducible chimeras, within an ectopically integrated transposon, in which the N-terminal (NTD) and C-terminal (CTD) domains of PilT and PilU have been swapped i.e. PilTN-PilUC (araC PBAD-pilTN-pilUC; TnpilTN-pilUC) and PilUN-PilTC (araC PBAD-pilUN-pilTC; TnpilUN-pilTC), were tested for functionality using (B) natural transformation as a readout for DNA-uptake pilus function and (C) surface motility as a readout for MSHA pilus function. To avoid interference, all variants were tested in a ΔpilTU background. The corresponding parental strains without a transposon served as negative controls. (A) The schematic illustrates the construction of the domain swapped PilT-PilU chimeras. The numbers in parentheses denote the source amino acid numbers of each domain. (B) Chitin-dependent transformation assay. Transformation frequencies are the mean of three repeats (+S.D.). < d.l., below detection limit. All strains were cultured on chitin in the presence of arabinose. The ability of TnpilT to complement the transformation phenotype of A1552ΔpilTU served as a positive control. (C) Surface motility was determined on soft LB agar plates, in the absence (- Ara) and presence (+ Ara) of induction, as indicated. The swarming diameter (cm) is the mean of three repeats (±S.D.). The ability of TnpilT to complement the gain of motility phenotype of A1552ΔpilTU served as a positive control. (PDF) [file pgen.1008393.s006.pdf]
