# Supplementary material for: The type IV pilus protein PilU functions as a PilT-dependent retraction ATPase
Source: PLoS Genet. 2019 Sep 16;15(9):e1008393. doi: 10.1371/journal.pgen.1008393 (PMC6762196; doi:10.1371/journal.pgen.1008393)
Supplement: S2 Fig — PilT and PilU protein sequences were aligned using Clustal Omega and the figure prepared using Jalview. Residues are shaded in graduations of blue according to sequence identity. Regions corresponding to the conserved Walker A box, Asp box, Walker B box and His box are highlighted in red. The conserved AIRNLIRE motif in PilT is also highlighted. A dashed red box indicates the residue (R206) that is substituted in V. cholerae strain MO10. Species abbreviations: [Vc]; Vibrio cholerae (CP028894.1), [Pa]; Pseudomonas aeruginosa (NC_002516.2), [Ps]; Pseudomonas stutzeri (CAB56295.1 and CAB56296.1), [Nm]; Neisseria meningitidis (FM999788.1), [Ng]; Neisseria gonorrhoeae (NC_002946.2), [Dn]; Dichelobacter nodosus (CP000513.1), [Ab]; Acinetobacter baylyi (NC_005966.1), [Aa]; Aquifex aeolicus (NC_000918.1), [Mx]; Myxococcus xanthus (NC_008095.1). Note that for simplicity, because M. xanthus has four additional PilT paralogues, only the experimentally validated PilT was included. (PDF) [file pgen.1008393.s002.pdf]

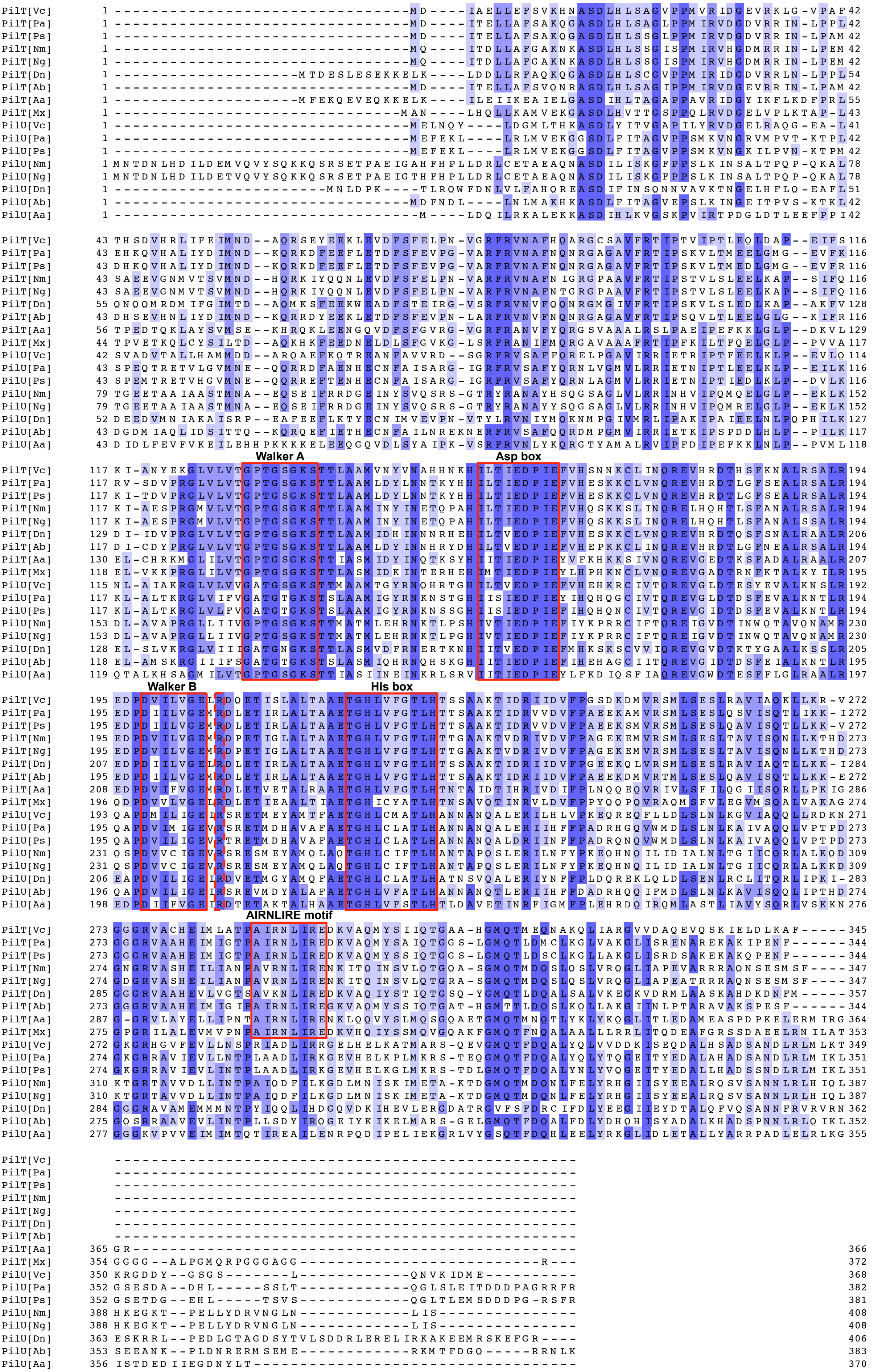

Walker A
Asp box
Walker B
His box
AIRNLIRE motif
