# Supplementary material for: The type IV pilus protein PilU functions as a PilT-dependent retraction ATPase
Source: PLoS Genet. 2019 Sep 16;15(9):e1008393. doi: 10.1371/journal.pgen.1008393 (PMC6762196; doi:10.1371/journal.pgen.1008393)
Supplement: S7 Fig — (A-C) PilT and PilU protein sequences from V. cholerae strains A1552 and MO10 were aligned using Clustal Omega and the figure prepared using Jalview. MO10 PilT and PilU sequences were identified using the MO10 genome sequence, GCA_000152425.1. (A) Alignments highlighting the conserved Walker A and Walker B motifs of PilT and PilU from strains A1552 and MO10. Residues are shaded in graduations of blue according to sequence identity. The R206S substitution present in PilT[MO10] is boxed in red. (B-C) Full-length alignments demonstrating that (B) PilT and (C) PilU are otherwise identical. (PDF) [file pgen.1008393.s007.pdf]

A

```
                          Walker A                              Walker B
PilT[A1552]   127  LVTGPTGSGKSTTL  140          195  EDPDVILVGELRD  207
PilT[MO10]    127  LVTGPTGSGKSTTL  140          195  EDPDVILVGELSD  207
PilU[A1552]   125  LVVGATGSGKSTTM  138          193  QAPDMILIGEIRS  205
PilU[MO10]    125  LVVGATGSGKSTTM  138          193  QAPDMILIGEIRS  205
```

B

```
PilT[A1552]     1  MDIAELLEFSVKHNASDLHLSAGVPPMVRIDGEVRKLGVPAFTH  44
PilT[MO10]      1  MDIAELLEFSVKHNASDLHLSAGVPPMVRIDGEVRKLGVPAFTH  44

PilT[A1552]    45  SDVHRLIFEIMNDAQRSEYEEKLEVDFSFELPNVGRFRVNAFHQ  88
PilT[MO10]     45  SDVHRLIFEIMNDAQRSEYEEKLEVDFSFELPNVGRFRVNAFHQ  88

PilT[A1552]    89  ARGCSAVFRTIPTVIPTLEQLDAPEIFSKIANYEKGLVLVTGPT  132
PilT[MO10]     89  ARGCSAVFRTIPTVIPTLEQLDAPEIFSKIANYEKGLVLVTGPT  132

PilT[A1552]   133  GSGKSTTLAAMVNYVNAHHNKHILTIEDPIEFVHSNNKCLINQR  176
PilT[MO10]    133  GSGKSTTLAAMVNYVNAHHNKHILTIEDPIEFVHSNNKCLINQR  176

PilT[A1552]   177  EVHRDTHSFKNALRSALREDPDVILVGELRDQETISLALTAAET  220
PilT[MO10]    177  EVHRDTHSFKNALRSALREDPDVILVGELSDQETISLALTAAET  220

PilT[A1552]   221  GHLVFGTLHTSSAAKTIDRIIDVFPGSDKDMVRSMLSESLRAVI  264
PilT[MO10]    221  GHLVFGTLHTSSAAKTIDRIIDVFPGSDKDMVRSMLSESLRAVI  264

PilT[A1552]   265  AQKLLKRVGGGRVACHEIMLATPAIRNLIREDKVAQMYSIIQTG  308
PilT[MO10]    265  AQKLLKRVGGGRVACHEIMLATPAIRNLIREDKVAQMYSIIQTG  308

PilT[A1552]   309  AAHGMQTMEQNAKQLIARGVVDAQEVQSKIELDLKAF  345
PilT[MO10]    309  AAHGMQTMEQNAKQLIARGVVDAQEVQSKIELDLKAF  345
```

C

```
PilU[A1552]     1  MELNQYLDGMLTHKASDLYITVGAPILYRVDGELRAQGEALSVA  44
PilU[MO10]      1  MELNQYLDGMLTHKASDLYITVGAPILYRVDGELRAQGEALSVA  44

PilU[A1552]    45  DVTALLHAMMDDARQAEFKQTREANFAVVRDSGRFRVSAFFQRE  88
PilU[MO10]     45  DVTALLHAMMDDARQAEFKQTREANFAVVRDSGRFRVSAFFQRE  88

PilU[A1552]    89  LPGAVIRRIETRIPTFEELKLPEVLQNLAIAKRGLVLVVGATGS  132
PilU[MO10]     89  LPGAVIRRIETRIPTFEELKLPEVLQNLAIAKRGLVLVVGATGS  132

PilU[A1552]   133  GKSTTMAAMTGYRNQHRTGHILTVEDPIEFVHEHKRCIVTQREV  176
PilU[MO10]    133  GKSTTMAAMTGYRNQHRTGHILTVEDPIEFVHEHKRCIVTQREV  176

PilU[A1552]   177  GLDTESYEVALKNSLRQAPDMILIGEIRSRETMEYAMTFAETGH  220
PilU[MO10]    177  GLDTESYEVALKNSLRQAPDMILIGEIRSRETMEYAMTFAETGH  220

PilU[A1552]   221  LCMATLHANNANQALERILHLVPKEQREQFLLDLSLNLKGVIAQ  264
PilU[MO10]    221  LCMATLHANNANQALERILHLVPKEQREQFLLDLSLNLKGVIAQ  264

PilU[A1552]   265  QLLRDKNGKGRHGVFEVLLNSPRIADLIRRGELHELKATMARSQ  308
PilU[MO10]    265  QLLRDKNGKGRHGVFEVLLNSPRIADLIRRGELHELKATMARSQ  308

PilU[A1552]   309  EVGMQTFDQALYQLVVDDKISEQDALHSADSANDLRLMLKTKRG  352
PilU[MO10]    309  EVGMQTFDQALYQLVVDDKISEQDALHSADSANDLRLMLKTKRG  352

PilU[A1552]   353  DDYGSGSLQNVKIDME  368
PilU[MO10]     353  DDYGSGSLQNVKIDME  368
```
