# Supplementary material for: The type IV pilus protein PilU functions as a PilT-dependent retraction ATPase
Source: PLoS Genet. 2019 Sep 16;15(9):e1008393. doi: 10.1371/journal.pgen.1008393 (PMC6762196; doi:10.1371/journal.pgen.1008393)
Supplement: S1 Table — (DOCX) [file pgen.1008393.s008.docx]

**S1 Table – Bacterial strains and plasmids.**

| **Name** | **GC#** | **Genotype**^a, b, c, d^ | **Source** |
| --- | --- | --- | --- |
| ***V. cholerae*** |  |  |  |
| A1552 | 1 | Wild type (WT), O1 El Tor Inaba; Rif^R^ | ([Yildiz & Schoolnik, 1998](#_ENREF_15)) |
| A1552∆*pilA* | 9 | ∆*pilA* | ([Meibom *et al.*, 2005](#_ENREF_7)) |
| A1552∆*pilB* | 10 | ∆*pilB* | ([Meibom *et al.*, 2005](#_ENREF_7)) |
| A1552∆*comEA* | 52 | ∆*comEA* | ([Meibom *et al.*, 2005](#_ENREF_7)) |
| A1552-*lacZ*-kan | 135 | Ω*lacZ*::*aph*; Kan^R^ | ([Marvig & Blokesch, 2010](#_ENREF_6)) |
| A1552-Tn*tfoX* | 1626 | Tn*tfoX* | ([Lo Scrudato & Blokesch, 2012](#_ENREF_5)) |
| A1552-Tn*tfoX*, ∆*pilT* | 2349 | Tn*tfoX*, ∆*pilT*::FRT | ([Seitz & Blokesch, 2013](#_ENREF_10)) |
| A1552-Tn*tfoX*, ∆*pilU* | 6152 | Tn*tfoX*, ∆*pilU*::FRT | This work |
| A1552-Tn*tfoX*, ∆*pilTU* | 6164 | Tn*tfoX*, ∆*pilTU*::FRT | This work |
| A1552-Tn*tfoX*, PilT[WA] | 6189 | Tn*tfoX*, *pilT*[K136A] | This work |
| A1552-Tn*tfoX*, PilT[WB] | 6157 | Tn*tfoX*, *pilT*[E204A] | This work |
| A1552-Tn*tfoX*, PilU[WA] | 6190 | Tn*tfoX*, *pilU*[K134A] | This work |
| A1552-Tn*tfoX*, PilU[WB] | 6192 | Tn*tfoX*, *pilU*[E202A] | This work |
| A1552-Tn*tfoX*, PilT[WA], ∆*pilU* | 6194 | Tn*tfoX*, *pilT*[K136A], ∆*pilU*::FRT | This work |
| A1552-Tn*tfoX*, PilT[WA], PilU[WA] | 6195 | Tn*tfoX*, *pilT*[K136A], *pilU*[K134A] | This work |
| A1552-Tn*tfoX*, PilT[WA], PilU[WB] | 6196 | Tn*tfoX*, *pilT*[K136A], *pilU*[E202A] | This work |
| A1552-Tn*tfoX*, PilT[WB], ∆*pilU* | 6165 | Tn*tfoX*, *pilT*[E204A], ∆*pilU*::FRT | This work |
| A1552-Tn*tfoX*, PilT[WB], PilU[WA] | 6191 | Tn*tfoX*, *pilT*[E204A], *pilU*[K134A] | This work |
| A1552-Tn*tfoX*, PilT[WB], PilU[WB] | 6193 | Tn*tfoX*, *pilT*[E204A], *pilU*[E202A] | This work |
| A1552-Tn*tfoX*, ∆*flaA* | 2000 | Tn*tfoX*, ∆*flaA*::FRT | ([Jaskólska *et al.*, 2018](#_ENREF_4)) |
| A1552-Tn*tfoX*, ∆*mshA* | 2822 | Tn*tfoX*, ∆*mshA*::FRT | ([Adams *et al.*, 2019](#_ENREF_1)) |
| A1552∆*flaA* | 1996 | ∆*flaA*::FRT | ([Van der Henst *et al.*, 2018](#_ENREF_12)) |
| A1552∆*mshA* | 2200 | ∆*mshA*::FRT | ([Seitz & Blokesch, 2013](#_ENREF_10)) |
| A1552∆*pilU* | 2353 | ∆*pilU*::FRT | ([Seitz & Blokesch, 2013](#_ENREF_10)) |
| A1552∆*pilT* | 2355 | ∆*pilT*::FRT | ([Seitz & Blokesch, 2013](#_ENREF_10)) |
| A1552-MshA[T70C] | 7819 | *mshA*[T70C] | This work |
| A1552-MshA[T70C], ∆*pilT* | 7914 | *mshA*[T70C] ∆*pilT*::FRT | This work |
| A1552-MshA[T70C], ∆*pilU* | 7915 | *mshA*[T70C] ∆*pilU*::FRT | This work |
| A1552-Tn*tfoX*, PilT[WA], MshA[T70C] | 7859 | Tn*tfoX*, *pilT*[K136A], *mshA*[T70C] | This work |
| A1552-Tn*tfoX*, PilT[WB], MshA[T70C] | 7860 | Tn*tfoX*, *pilT*[E204A], *mshA*[T70C] | This work |
| A1552-Tn*tfoX*, PilU[WA], MshA[T70C] | 7861 | Tn*tfoX*, *pilU*[K134A], *mshA*[T70C] | This work |
| A1552-Tn*tfoX*, PilU[WB], MshA[T70C] | 7862 | Tn*tfoX*, *pilU*[E202A], *mshA*[T70C] | This work |
| A1552-Tn*tfoX*, PilT[WA], ∆*pilU*, MshA[T70C] | 7863 | Tn*tfoX*, *pilT*[K136A], ∆*pilU*::FRT, *mshA*[T70C] | This work |
| A1552-Tn*tfoX*, PilT[WA], PilU[WA], MshA[T70C] | 7864 | Tn*tfoX*, *pilT*[K136A], *pilU*[K134A], *mshA*[T70C] | This work |
| A1552-Tn*tfoX*, PilT[WA], PilU[WB], MshA[T70C] | 7865 | Tn*tfoX*, *pilT*[K136A], *pilU*[E202A], *mshA*[T70C] | This work |
| A1552-Tn*tfoX*, PilT[WB], ∆*pilU*, MshA[T70C] | 7866 | Tn*tfoX*, *pilT*[E204A], ∆*pilU*::FRT, *mshA*[T70C] | This work |
| A1552-Tn*tfoX*, PilT[WB], PilU[WA], MshA[T70C] | 7867 | Tn*tfoX*, *pilT*[E204A], *pilU*[K134A], *mshA*[T70C] | This work |
| A1552-Tn*tfoX*, PilT[WB], PilU[WB], MshA[T70C] | 7868 | Tn*tfoX*, *pilT*[E204A], *pilU*[E202A], *mshA*[T70C] | This work |
| A1552-Tn*tfoX*, PilA[S67C] | 5811 | Tn*tfoX*, *pilA*[S67C] | ([Adams *et al.*, 2019](#_ENREF_1)) |
| A1552-Tn*tfoX*, PilA[S67C], ∆*pilT* | 6178 | Tn*tfoX*, *pilA*[S67C], ∆*pilT*::FRT | ([Adams *et al.*, 2019](#_ENREF_1)) |
| A1552-Tn*tfoX*, PilA[S67C], ∆*pilU* | 6187 | Tn*tfoX*, *pilA*[S67C], ∆*pilU*::FRT | ([Adams *et al.*, 2019](#_ENREF_1)) |
| A1552-Tn*tfoX*, PilT[WA], PilA[S67C] | 7847 | Tn*tfoX*, *pilT*[K136A], *pilA*[S67C] | This work |
| A1552-Tn*tfoX*, PilT[WB], PilA[S67C] | 7848 | Tn*tfoX*, *pilT*[E204A], *pilA*[S67C] | This work |
| A1552-Tn*tfoX*, PilU[WA], PilA[S67C] | 7849 | Tn*tfoX*, *pilU*[K134A], *pilA*[S67C] | This work |
| A1552-Tn*tfoX*, PilU[WB], PilA[S67C] | 7850 | Tn*tfoX*, *pilU*[E202A], *pilA*[S67C] | This work |
| A1552-Tn*tfoX*, PilT[WA], ∆*pilU*, PilA[S67C] | 7851 | Tn*tfoX*, *pilT*[K136A], ∆*pilU*::FRT, *pilA*[S67C] | This work |
| A1552-Tn*tfoX*, PilT[WA], PilU[WA], PilA[S67C] | 7852 | Tn*tfoX*, *pilT*[K136A], *pilU*[K134A], *pilA*[S67C] | This work |
| A1552-Tn*tfoX*, PilT[WA], PilU[WB], PilA[S67C] | 7853 | Tn*tfoX*, *pilT*[K136A], *pilU*[E202A], *pilA*[S67C] | This work |
| A1552-Tn*tfoX*, PilT[WB], ∆*pilU*, PilA[S67C] | 7854 | Tn*tfoX*, *pilT*[E204A], ∆*pilU*::FRT, *pilA*[S67C] | This work |
| A1552-Tn*tfoX*, PilT[WB], PilU[WA], PilA[S67C] | 7855 | Tn*tfoX*, *pilT*[E204A], *pilU*[K134A], pilA[S67C] | This work |
| A1552-Tn*tfoX*, PilT[WB], PilU[WB], PilA[S67C] | 7856 | Tn*tfoX*, *pilT*[E204A], *pilU*[E202A], *pilA*[S67C] | This work |
| A1552-PilT-3xFLAG | 8368 | *pilT*-3xFLAG | This work |
| A1552-PilU-3xFLAG | 7931 | *pilU*-3xFLAG | This work |
| A1552∆*pilT*, PilU-3xFLAG | 7932 | ∆*pilT*::FRT, *pilU*-3xFLAG | This work |
| A1552-  Tn*pilU*-3xFLAG | 8292 | Tn*pilU*-3xFLAG | This work |
| A1552∆*pilT*, Tn*pilT* | 7886 | ∆*pilT*::FRT, Tn*pilT* | This work |
| A1552∆*pilU*, Tn*pilU* | 7887 | ∆*pilU*::FRT, Tn*pilU* | This work |
| A1552∆*pilT*, Tn*pilU* | 7888 | ∆*pilT*::FRT, Tn*pilU* | This work |
| A1552-PilT[WA], ∆*pilU* | 7927 | *pilT*[K136A], ∆*pilU*::FRT | This work |
| A1552-PilT[WA], ∆*pilU*, Tn*pilU* | 7971 | *pilT*[K136A], ∆*pilU*::FRT, Tn*pilU* | This work |
| A1552-PilT[WB], ∆*pilU* | 7928 | *pilT*[E204A], ∆*pilU*::FRT | This work |
| A1552-PilT[WB], ∆*pilU*, Tn*pilU* | 7972 | *pilT*[E204A], ∆*pilU*::FRT, Tn*pilU* | This work |
| A1552∆*pilU*, Tn*pilT* | 8280 | ∆*pilU*::FRT, Tn*pilT* | This work |
| A1552∆*pilU*, Tn*pilT*[WA] | 8293 | ∆*pilU*::FRT, Tn*pilT*[K136A] | This work |
| A1552∆*pilU*, Tn*pilT*[WB] | 8281 | ∆*pilU*::FRT, Tn*pilT*[E204A] | This work |
| A1552∆*pilU*, Tn*pilU*[WA] | 8282 | ∆*pilU*::FRT, Tn*pilU*[K134A] | This work |
| A1552∆*pilU*, Tn*pilU*[WB] | 8283 | ∆*pilU*::FRT, Tn*pilU*[E202A] | This work |
| A1552  ∆*pilTU*::FRT | 8387 | ∆*pilTU*::FRT | This work |
| A1552  ∆*pilTU*::FRT, Tn*pilT* | 8362 | ∆*pilTU*::FRT, Tn*pilT* | This work |
| A1552  ∆*pilTU*::FRT, Tn*pilU* | 8363 | ∆*pilTU*::FRT, Tn*pilU* | This work |
| A1552  ∆*pilTU*::FRT, Tn*pilT*^N^-*pilU*^C^ | 8364 | ∆*pilTU*::FRT, Tn*pilT*^N^-*pilU*^C^ | This work |
| A1552  ∆*pilTU*::FRT, Tn*pilU*^N^-*pilT*^C^ | 8365 | ∆*pilTU*::FRT, Tn*pilU*^N^-*pilT*^C^ | This work |
| MO10 | 5 | MO10; O139 strain; isolated in 1992, India; *hapR* mutated (HapR[R12L]), Str^R^ | ([Waldor & Mekalanos, 1994a](#_ENREF_13), [Waldor & Mekalanos, 1994b](#_ENREF_14)) |
| MO10-*hapR*^Rep^ | 5661 | MO10-*hapR*^Rep^ (HapR[R12L] variant repaired) | This work |
| MO10-Tn*tfoX* | 5924 | MO10-Tn*tfoX* | This work |
| MO10-*hapR*^Rep^, Tn*tfoX* | 5690 | MO10-*hapR*^Rep^, Tn*tfoX* | This work |
| MO10-Tn*tfoX*, ∆*pilA* | 6002 | MO10-Tn*tfoX*, ∆*pilA* | This work |
| MO10-*hapR*^Rep^, Tn*tfoX*, ∆*pilA* | 5940 | MO10-*hapR*^Rep^, Tn*tfoX*, ∆*pilA* | This work |
| MO10-Tn*tfoX*, ∆*pilT* | 5970 | MO10-Tn*tfoX*, ∆*pilT* | This work |
| MO10-*hapR*^Rep^, Tn*tfoX*, ∆*pilT* | 5971 | MO10-*hapR*^Rep^, Tn*tfoX*, ∆*pilT* | This work |
| MO10-Tn*tfoX*, *pilT*[A1552] | 6018 | MO10-Tn*tfoX*, *pilT*[PilT A1552] | This work |
| MO10-*hapR*^Rep^, Tn*tfoX*, *pilT*[A1552] | 6019 | MO10-*hapR*^Rep^, Tn*tfoX*, *pilT*[PilT A1552] | This work |
| MO10-Tn*tfoX*, PilA[S67C] | 6029 | MO10-Tn*tfoX*, *pilA*[S67C] | This work |
| MO10-*hapR*^Rep^, Tn*tfoX*, PilA[S67C] | 5964 | MO10-*hapR*^Rep^, Tn*tfoX*, *pilA*[S67C] | This work |
| MO10-*hapR*^Rep^, Tn*tfoX*, PilA[S67C],  *pilT*[A1552] | 7858 | MO10-*hapR*^Rep^, Tn*tfoX*, *pilT*[PilT A1552], *pilA*[S67C] | This work |
| MO10-MshA[T70C] | 7869 | MO10-*mshA*[T70C] | This work |
| MO10-MshA[T70C],  *pilT*[A1552] | 7871 | MO10-*pilT*[PilT A1552], *mshA*[T70C] | This work |
| MO10-*hapR*^Rep^, Tn*tfoX*, ∆*pilU* | 8376 | MO10-*hapR*^Rep^, Tn*tfoX*, ∆*pilU* | This work |
| MO10-*hapR*^Rep^, Tn*tfoX*, *pilT*[PilT A1552], ∆*pilU* | 8377 | MO10-*hapR*^Rep^, Tn*tfoX*, *pilT*[PilT A1552], ∆*pilU* | This work |
| MO10-MshA[T70C], ∆*pilU* | 8378 | MO10-*mshA*[T70C], ∆*pilU* | This work |
| A1552-Tn*tfoX*, *pilT*[MO10] | 5968 | Tn*tfoX*, *pilT*[PilT MO10] | This work |
| A1552-Tn*tfoX*, *pilT*[MO10], ∆*pilU* | 6197 | Tn*tfoX*, *pilT*[PilT MO10], ∆*pilU*::FRT | This work |
| A1552- *pilT*[MO10] | 5967 | *pilT*[PilT MO10] | This work |
| A1552∆*pilT*, Tn*pilT*[Pa] | 8249 | ∆*pilT*::FRT, Tn*pilT*[Pa] | This work |
| A1552∆*pilT*, Tn*pilU*[Pa] | 8250 | ∆*pilT*::FRT, Tn*pilU*[Pa] | This work |
| A1552∆*pilU*, Tn*pilU*[Pa] | 8251 | ∆*pilU*::FRT, Tn*pilU*[Pa] | This work |
| A1552-PilT[WA], ∆*pilU*, Tn*pilU*[Pa] | 8252 | *pilT*[K136A], ∆*pilU*::FRT, Tn*pilU*[Pa] | This work |
| A1552-PilT[WB], ∆*pilU*, Tn*pilU*[Pa] | 8253 | *pilT*[E204A], ∆*pilU*::FRT, Tn*pilU*[Pa] | This work |
| ***E. coli*** |  |  |  |
| S17 λpir | 648 | Tp^r^ Sm^r^ *recA thi pro hsdR-M+ RP4:2-Tc:Mu:*  Km^r^ Tn*7* (λ*pir*) | ([Simon *et al.*, 1983](#_ENREF_11)) |
| XL10 gold | 734 | Tet^r^∆(*mcrA*)*183* ∆(*mcrCB-hsdSMR-mrr*)*173 endA1 supE44 thi-1 recA1 gyrA96 relA1 lac* Hte [F ́ *proAB lacI*^q^*Z*∆*M15* Tn*10* (Tet^r^) Amy Cam^r^]. | Stratagene;  Cat# 200315. |
| BTH101 | 6221 | F-, *cya-99*, *araD139, galE15, galK16, rpsL1* (Str^r^), *hsdR2, mcrA1, mcrB1.* | Euromedex; EUB001 |
| **Plasmids** |  |  |  |
| pBR-FRT-Kan-FRT2 | 3782 | pBR322 derivative containing improved FRT-*aph*-FRT cassette, used as template for TransFLP; Amp^R^, Kan^R^ | ([Metzger *et al.*, 2016](#_ENREF_9)) |
| pBR-FLP | 1203 | pBR322 derivative containing FLP+, λ cI857+, λ pR from pCP20 integrated into the *EcoRV* site of pBR322, used for FLP recombination; Amp^R^ | ([De Souza Silva & Blokesch, 2010](#_ENREF_3)) |
| pUX-BF13 | 457 | pUX-BF13 - oriR6K, helper plasmid with Tn7 transposition function; Amp^R^ | ([Bao *et al.*, 1991](#_ENREF_2)) |
| pGP704-mTn*tfoX* | 1624 | pGP704 with mini-Tn7 carrying *araC* and *P*_BAD_-*tfoX*; Amp^R^, Gent^R^ (Tn*tfoX*) | ([Lo Scrudato & Blokesch, 2012](#_ENREF_5)) |
| p28-*pilA* | 1050 | pGP704-Sac28-∆*pilA* | ([Meibom *et al.*, 2004](#_ENREF_8)) |
| p28-*mshA* | 1051 | pGP704-Sac28-∆*mshA* | ([Meibom *et al.*, 2004](#_ENREF_8)) |
| p28-PilA[S67C] | 6202 | pGP704-Sac28-*pilA*[S67C] | ([Adams *et al.*, 2019](#_ENREF_1)) |
| p28-MshA[T70C] | 7818 | pGP704-Sac28-*mshA*[T70C] | This work |
| p28-PilT[K136A] | 6209 | pGP704-Sac28-*pilT*[K136A] | This work |
| p28-PilT[E204A] | 6198 | pGP704-Sac28-*pilT*[E204A] | This work |
| p28-PilU[K134A] | 6210 | pGP704-Sac28-*pilU*[K134A] | This work |
| p28-PilU[E202A] | 6211 | pGP704-Sac28-*pilU*[E202A] | This work |
| pGP704-*hapR*^Rep^ | 5648 | pGP704-Sac28-*hapR*^Rep^ (repairs *hapR* to the A1552 sequence) | ([Van der Henst *et al.*, 2018](#_ENREF_12)) |
| p28-∆*pilT* | 5959 | pGP704-Sac28-∆*pilT* | ([Adams *et al.*, 2019](#_ENREF_1)) |
| p28-∆*pilU* | 8367 | pGP704Sac28-∆*pilU* | This work |
| p28-PilT[A1552] | 5960 | pGP704-Sac28-*pilT*[A1552] | This work |
| p28-PilT[MO10] | 5961 | pGP704-Sac28-*pilT*[MO10] | This work |
| p28-PilT-3xFLAG | 8366 | pGP704Sac28-*pilT*-3xFLAG | This work |
| p28-PilU-3xFLAG | 7916 | pGP704-Sac28-*pilU*-3xFLAG | This work |
| pGP704-mTn*pilT* | 8284 | pGP704 with mini-Tn7 carrying *araC* and *P*_BAD_-*pilT*; Amp^R^, Gent^R^ (Tn*pilT*) | This work |
| pGP704-mTn*pilU* | 8285 | pGP704 with mini-Tn7 carrying *araC* and *P*_BAD_-*pilU*; Amp^R^, Gent^R^ (Tn*pilU*) | This work |
| pGP704-mTn-*pilU*-3xFLAG | 8267 | pGP704 with mini-Tn7 carrying *araC* and *P*_BAD_-*pilU*-3xFLAG; Amp^R^, Gent^R^ (Tn*pilU*-3xFLAG) | This work |
| pGP704-mTn-*pilT*[K136A] | 8268 | pGP704 with mini-Tn7 carrying *araC* and *P*_BAD_-*pilT*[K136A]; Amp^R^, Gent^R^ (Tn*pilT*[K136A]) | This work |
| pGP704-mTn-*pilT*[E204A] | 8269 | pGP704 with mini-Tn7 carrying *araC* and *P*_BAD_-*pilT*[E204A]; Amp^R^, Gent^R^ (Tn*pilT*[E204A]) | This work |
| pGP704-mTn-*pilU*[K134A] | 8270 | pGP704 with mini-Tn7 carrying *araC* and *P*_BAD_-*pilU*[K134A]; Amp^R^, Gent^R^ (Tn*pilU*[K134A]) | This work |
| pGP704-mTn-*pilU*[E202A] | 8271 | pGP704 with mini-Tn7 carrying *araC* and *P*_BAD_-*pilU*[E202A]; Amp^R^, Gent^R^ (Tn*pilU*[E202A]) | This work |
| pGP704-mTn- Tn*pilT*^N^-*pilU*^C^ | 8312 | pGP704 with mini-Tn7 carrying *araC* and *P*_BAD_-*pilT*^N^-*pilU*^C^; Amp^R^, Gent^R^ (Tn*pilT*^N^-*pilU*^C^) | This work |
| pGP704-mTn- Tn*pilU*^N^-*pilT*^C^ | 8313 | pGP704 with mini-Tn7 carrying *araC* and *P*_BAD_-*pilU*^N^-*pilT*^C^; Amp^R^, Gent^R^ (Tn*pilU*^N^-*pilT*^C^) | This work |
| pGP704-mTn*pilT*[Pa] | 8233 | pGP704 with mini-Tn7 carrying *araC* and *P*_BAD_-*pilT*[Pa]; Amp^R^, Gent^R^ (Tn*pilT*[Pa]).  Pa = *P. aeruginosa* strain PAO1. | This work |
| pGP704-mTn*pilU*[Pa] | 8234 | pGP704 with mini-Tn7 carrying *araC* and *P*_BAD_-*pilU*[Pa]; Amp^R^, Gent^R^ (Tn*pilU*[Pa]).  Pa = *P. aeruginosa* strain PAO1. | This work |
| pUT18 | 6222 | pUT18; BACTH C-terminal T18 fusions, Amp^R^ | Euromedex; EUP-18N |
| pUT18C | 6223 | pUT18C; BACTH N-terminal T18 fusions, Amp^R^ | Euromedex; EUP-18C |
| pUT18C-zip | 6224 | T18-leucine zipper from yeast GCN4; Amp^R^ | Euromedex; EUP-18Z |
| pKT25 | 6225 | pKT25; BACTH N-terminal T25 fusions, Kan^R^ | Euromedex; EUP-25C |
| pKNT25 | 6226 | pKNT25; BACTH C-terminal T25 fusions, Kan^R^ | Euromedex; EUP-25N |
| pKT25-zip | 6227 | T25-leucine zipper from yeast GCN4; Kan^R^ | Euromedex; EUP-25Z |
| pUT18-*pilT* | 6213 | pUT18-*pilT* (PilT-T18); Amp^R^ | This work |
| pUT18C-*pilT* | 6214 | pUT18C-*pilT* (T18-PilT); Amp^R^ | This work |
| pKT25-*pilT* | 6215 | pKT25-*pilT* (T25-PilT); Kan^R^ | This work |
| pKNT25-*pilT* | 6216 | pKNT25-*pilT* (PilT-T25); Kan^R^ | This work |
| pUT18-*pilU* | 6217 | pUT18-*pilU* (PilU-T18); Amp^R^ | This work |
| pUT18C-*pilU* | 6218 | pUT18C-*pilU* (T18-PilU); Amp^R^ | This work |
| pKT25-*pilU* | 6219 | pKT25-*pilU* (T25-PilU); Kan^R^ | This work |
| pKNT25-*pilU* | 6220 | pKNT25-*pilU* (PilU-T25); Kan^R^ | This work |
| pUT18-*pilB* | 7889 | pUT18-*pilB* (PilB-T18); Amp^R^ | This work |
| pUT18C-*pilB* | 7890 | pUT18C-*pilB* (T18-PilB); Amp^R^ | This work |
| pKT25-*pilB* | 7891 | pKT25-*pilB* (T25-PilB); Kan^R^ | This work |
| pKNT25-*pilB* | 7892 | pKNT25-*pilB* (PilB-T25); Kan^R^ | This work |

^a^ Unless stated otherwise, all *V. cholerae* strains are derivatives of A1552 (GC#1; Rif^R^).

^b^ Strains containing mini-Tn7 insertions (*i.e.* Tn*tfoX*, Tn*pilT*, Tn*pilU*) are Gent^R^.

^c^ Derivatives of MO10 (GC#5) are Str^R^.

^d^ FRT; flippase recognition target (FRT) scar left behind by TransFLP method.

**Supporting references**

Adams, D.W., S. Stutzmann, C. Stoudmann & M. Blokesch, (2019) DNA-uptake pili of *Vibrio cholerae* are required for chitin colonization and capable of kin recognition via sequence-specific self-interaction. *Nature microbiology* **4**: 1545-1557.

Bao, Y., D.P. Lies, H. Fu & G.P. Roberts, (1991) An improved Tn7-based system for the single-copy insertion of cloned genes into chromosomes of gram-negative bacteria. *Gene* **109**: 167-168.

De Souza Silva, O. & M. Blokesch, (2010) Genetic manipulation of *Vibrio cholerae* by combining natural transformation with FLP recombination. *Plasmid* **64**: 186-195.

Jaskólska, M., S. Stutzmann, C. Stoudmann & M. Blokesch, (2018) QstR-dependent regulation of natural competence and type VI secretion in *Vibrio cholerae*. *Nucleic acids research* **46**: 10619-10634.

Lo Scrudato, M. & M. Blokesch, (2012) The regulatory network of natural competence and transformation of *Vibrio cholerae*. *PLoS genetics* **8**: e1002778.

Marvig, R.L. & M. Blokesch, (2010) Natural transformation of *Vibrio cholerae* as a tool - optimizing the procedure. *BMC microbiology* **10**: 155.

Meibom, K.L., M. Blokesch, N.A. Dolganov, C.Y. Wu & G.K. Schoolnik, (2005) Chitin induces natural competence in *Vibrio cholerae*. *Science* **310**: 1824-1827.

Meibom, K.L., X.B. Li, A.T. Nielsen, C.Y. Wu, S. Roseman & G.K. Schoolnik, (2004) The *Vibrio cholerae* chitin utilization program. *Proceedings of the National Academy of Sciences of the United States of America* **101**: 2524-2529.

Metzger, L.C., S. Stutzmann, T. Scrignari, C. Van der Henst, N. Matthey & M. Blokesch, (2016) Independent Regulation of Type VI Secretion in *Vibrio cholerae* by TfoX and TfoY. *Cell reports* **15**: 951-958.

Seitz, P. & M. Blokesch, (2013) DNA-uptake machinery of naturally competent *Vibrio cholerae*. *Proceedings of the National Academy of Sciences of the United States of America* **110**: 17987-17992.

Simon, R., U. Priefer & A. Pühler, (1983) A Broad Host Range Mobilization System for In Vivo Genetic Engineering: Transposon Mutagenesis in Gram Negative Bacteria. *Bio/Technology* **1**: 784.

Van der Henst, C., A.S. Vanhove, N.C. Drebes Dörr, S. Stutzmann, C. Stoudmann, S. Clerc, T. Scrignari, C. Maclachlan, G. Knott & M. Blokesch, (2018) Molecular insights into *Vibrio cholerae*’s intra-amoebal host-pathogen interactions. *Nature communications* **9**: 3460.

Waldor, M.K. & J.J. Mekalanos, (1994a) Emergence of a new cholera pandemic: molecular analysis of virulence determinants in *Vibrio cholerae* O139 and development of a live vaccine prototype. *The Journal of infectious diseases* **170**: 278-283.

Waldor, M.K. & J.J. Mekalanos, (1994b) ToxR regulates virulence gene expression in non-O1 strains of *Vibrio cholerae* that cause epidemic cholera. *Infection and immunity* **62**: 72-78.

Yildiz, F.H. & G.K. Schoolnik, (1998) Role of *rpoS* in stress survival and virulence of *Vibrio cholerae*. *Journal of bacteriology* **180**: 773-784.
